# Supplementary material for: Do the shuffle: Exploring reasons for music listening through shuffled play
Source: PLoS One. 2020 Feb 6;15(2):e0228457. doi: 10.1371/journal.pone.0228457 (PMC7004375; doi:10.1371/journal.pone.0228457)
Supplement: S1 File — Administered online through Qualtrics. (PDF) [file pone.0228457.s001.pdf]

Do the Shuffle: A research study

Start of Block: Intro/Consent

Q1.1
Welcome to Do the Shuffle!

This research study is about people's everyday music listening.

To participate you need to be: **Over the age of 18 Have your everyday music listening device/application handy, e.g. iPod, Spotify, iTunes, SoundCloud. The device/application must have a shuffle function If you use playlists make sure they are playlists that you have created yourself.**
The aim of the study is to gain a better understanding of people's everyday relationships with music. This will help us better understand how it may be used within community contexts to benefit people.

If you have already taken part in this study, we thank you. Please do not take this survey again.

This study is in three parts:
Part 1: Firstly, you will be asked to take out your regular music listening device (e.g. phone, laptop etc.), turn on the shuffle function within a listening app you usually use and press play. Whatever song comes on first (no matter which one it is), you will be invited to answer some open-ended questions about this song. You will then be asked to do this entire process again, but with a different song (using shuffle again).

Part 2: Next, you will answer some short multiple choice questions about the two songs you just listened to.

Part 3: Finally, you will be asked to fill out another short questionnaire about yourself and your music listening habits.

We expect this whole process to take around 15 minutes.

This study is organised by members of the research team at Nordoff Robbins, a music therapy charity, and the School of Psychology at Keele University.

If you have any questions about this study, please contact: research@nordoff-robbins.org.uk

If you have any concerns about this study, please contact: simon.procter@nordoff-robbins.org.uk

If you would like to participate please click the red button below to continue.

Page Break

Q71 I have taken part in this study, Do the Shuffle, before.

◯ Yes (1)

◯ No (2)

Q1.2 To make sure you are eligible and informed about the study please complete the following questions:

I confirm that I am over the age of 18.

◯ Yes (1)

◯ No (2)

Q1.3 I understand that this project is run by the Nordoff Robbins research team and is also part of an undergraduate dissertation at Keele University

◯ Yes (1)

◯ No (2)

Q1.4 I understand that if any personal information is within my responses the Nordoff Robbins/Keele University research team will not quote it.

◯ Yes (1)

◯ No (2)

Q1.5 I understand that I can withdraw from the study at any time, without any consequences.

- ◯ Yes (1)
- ◯ No (2)

Q1.6 I understand that all data will be kept confidential and kept securely.

- ◯ Yes (1)
- ◯ No (2)

Q1.7 I understand and accept that the data will be published in an article written by the researchers.

- ◯ Yes (1)
- ◯ No (2)

Q1.8 I give consent to take part in this study.

- ◯ Yes (1)
- ◯ No (2)

End of Block: Intro/Consent

Start of Block: Disqualification

Q58 We are sorry but you are not able to participant in this study.

We thank you for your interest. If you want to learn more about the study or follow its progress please visit our website: https://www.nordoff-robbins.org.uk/

Please click below to exit the questionnaire.

End of Block: Disqualification

Start of Block: Consent to be quoted

Q59 Great! You are almost ready to get started. We just want to ask a few questions about consent to be quoted

---

Q1.9 I give my consent to be quoted in an academic publication.

- ○ Yes (1)
- ○ No (2)

Q1.10 If yes, choose one:

- ○ Anonymously (1)
- ○ By the name/pseudonym you would like to use: (2) ____

Q1.11 I give my consent to be quoted on the Nordoff Robbins website.

- ○ Yes (1)
- ○ No (2)

Q1.12 If yes, choose one:

○ Anonymously (1)

○ By the name/pseudonym you would like to use: (2)
____

Q1.13 To start Do the Shuffle, please click the button below.

End of Block: Consent to be quoted

Start of Block: Song Selection (song 1)

Q2.1
PART ONE: Your First Shuffle
On your regular music listening device (e.g. phone, laptop etc.), turn on the shuffle function within your listening app and press play.

If you are using a playlist please ensure you are only using a playlist you have created yourself.

Whatever track comes on FIRST (no matter which one it is), please write that track title and artist below as well as the device/application you are using. You can choose to listen to the track while you answer the rest of the questions.

Q2.2 Track Title:

____

Q2.3 Artist:

____

Q2.4 Music Device/App:

Q2.5 What's the first thing that comes into your mind about this track?

Q2.6 Why did you choose to save this track to your device?

Q2.7 Do you enjoy listening to this track?

- ◯ Really do not enjoy (1)
- ◯ Do not enjoy (2)
- ◯ Neither enjoy/not enjoy (3)
- ◯ Enjoy (4)
- ◯ Really Enjoy (5)

Q2.8 Why do you enjoy or not enjoy listening to the track?

____

____

____

____

____

Q2.9 Do you have a relationship with this track? Whether it's 'Yes' or 'No' please explain.

____

____

____

____

____

Q2.10 We'd now like you to repeat part one so please press shuffle again and press the button below to continue.

End of Block: Song Selection (song 1)

Start of Block: Song Selection (song 2)

Q3.1
PART ONE: Your Second Shuffle

On your regular music listening device (e.g. phone, laptop etc.), turn on the shuffle function within your listening app and press play.

If you are using a playlist please ensure you are only using a playlist you have created yourself.

Whatever track comes on ***FIRST*** (no matter which one it is), please write that track title and artist below as well as the device/application you are using. You can choose to listen to the track while you answer the rest of the questions.

Q3.2 Track Title:

____

Q3.3 Artist:

____

Q3.4 Music Device/App

____

Q3.5 What's the first thing that comes into your mind about this track?

____

Q3.6 Why did you choose to save this track to your device?

Q3.7 Do you enjoy listening to this track?

- Really do not enjoy (1)
- Do not enjoy (2)
- Neither enjoy/not enjoy (3)
- Enjoy (4)
- Really Enjoy (5)

Q3.8 Why do you enjoy or not enjoy listening to the track?

Q3.9 Do you have a relationship with this track? Whether it's 'Yes' or 'No' please explain.

____

____

____

____

____

End of Block: Song Selection (song 2)

Start of Block: Part 2 (song 1)

Q4.1
PART TWO: Questions About Your First Shuffle
We are going to ask you more about the two tracks you just heard.
For the first track you listened to, ${Q2.2/ChoiceTextEntryValue}, please answer the following 3 questions.

Why do you usually listen to this track?

Please pick up to 5 reasons and rank them in order from most to least relevant to you. ***1 – most relevant, 5 – least relevant.***

______ To help me concentrate/think (1)
______ To distract me (2)
______ To help me relax (3)
______ To help me carry out/enhance the activity I was doing (4)
______ To help me create the right atmosphere (5)
______ To bring back certain memories (16)
______ To create an emotion or mood (6)
______ To accentuate an emotion/mood (7)
______ To change an existing emotion/mood (8)
______ Because I really like listening to it (9)
______ Because I thought the person(s) I was with would like it (10)
______ To help me pass the time (11)
______ To help me feel less alone (12)
______ To listen to the lyrics (13)
______ Out of habit (14)
______ None of the above (15)

Q4.2 Why did you download this track? Please select all that apply.

- ▢ It is one of my favourite tracks (1)
- ▢ It is a track that has strong meaning for me (2)
- ▢ I like the way it sounds (3)
- ▢ I heard it somewhere when I was out (e.g. at an event, at the cinema, in a pub) (4)
- ▢ I heard it on a playlist or on the radio (5)
- ▢ It was a recommendation from someone I know personally (6)
- ▢ It was a recommendation I heard or from a review I read (7)
- ▢ My application recommended it (8)
- ▢ It was advertised (9)
- ▢ It was free (10)
- ▢ I liked the album (11)
- ▢ I liked the artist (12)
- ▢ I liked the genre (13)
- ▢ I have never heard it before (14)
- ▢ Other: please specify (15) ____________________

Q4.3 When was the last time you heard **${Q2.2/ChoiceTextEntryValue}**?

- In the last two days (1)
- In the last week (2)
- In the last month (3)
- In the last 6 months (4)
- In the last year (5)
- Over a year ago (6)
- I have never heard this song (7)

End of Block: Part 2 (song 1)

Start of Block: Part 2 (song 2)

Q5.1

PART TWO: Questions About Your Second Shuffle

We are now going to ask you more about the second track you heard.

For the second track you heard, **${Q3.2/ChoiceTextEntryValue}**, please answer the following 3 questions.
Why do you usually listen to this track?

Please pick up to 5 reasons and rank them in order from most to least relevant to you. ***1 – most relevant, 5 – least relevant.***
______ To help me concentrate/think (1)
______ To distract me (2)
______ To help me relax (3)
______ To help me carry out/enhance the activity I was doing (4)
______ To help me create the right atmosphere (5)
______ To bring back certain memories (16)
______ To create an emotion or mood (6)
______ To accentuate an emotion/mood (7)
______ To change an existing emotion/mood (8)
______ Because I really like listening to it (9)
______ Because I thought the person(s) I was with would like it (10)
______ To help me pass the time (11)
______ To help me feel less alone (12)
______ To listen to the lyrics (13)
______ Out of habit (14)
______ None of the above (15)

Q5.2 Why did you download this track? Please select all that apply.

- ▢ It is one of my favourite tracks (1)
- ▢ It is a track that has strong meaning for me (2)
- ▢ I like the way it sounds (3)
- ▢ I heard it somewhere when I was out (e.g. at an event, at the cinema, in a pub) (4)
- ▢ I heard it on a playlist or on the radio (5)
- ▢ It was a recommendation from someone I know personally (6)
- ▢ It was a recommendation I heard or from a review I read (7)
- ▢ My application recommended it (8)
- ▢ It was advertised (9)
- ▢ It was free (10)
- ▢ I liked the album (11)
- ▢ I liked the artist (12)
- ▢ I liked the genre (13)
- ▢ I have never heard it before (14)
- ▢ Other: please specify (15) ________________________________

Q5.3 When was the last time you heard **${Q3.2/ChoiceTextEntryValue/1}**?

◯ In the last two days (1)

◯ In the last week (2)

◯ In the last month (3)

◯ In the last 6 months (4)

◯ In the last year (5)

◯ Over a year ago (6)

◯ I have never heard this track (7)

End of Block: Part 2 (song 2)

Start of Block: Part 3

Q69
PART THREE

Q6.1 How do you describe yourself? *(Optional)*

◯ Male (1)

◯ Female (2)

◯ Rather not say (3)

Q6.2 How old are you? *(Optional)*

▼ 18 (1) ... 100 (83)

Q69 In which country do you currently reside? *(Optional)*

▼ Afghanistan (1) ... Zimbabwe (1357)

Q70 Which country are you originally from? *(Optional)*

▼ Afghanistan (1) ... Zimbabwe (1357)

Q6.5 How do you usually listen to your music?

- ○ On my personal listening devices (through iTunes, Spotify, Soundcloud, etc.) (1)
- ○ Other (please specify): (2) ____

Q6.6 How do you usually choose the music you listen to?

- ○ Choose which tracks I want to listen to when I want to listen to them (1)
- ○ Use self-selected playlists (2)
- ○ Using playlists created by others (3)
- ○ Radio (4)
- ○ Other (please specify): (5) ____

Q6.7 I usually... (select all that apply)

- ☐ Buy my music online (1)
- ☐ Buy my music in a shop (2)
- ☐ Listen via a subscription to a site (e.g. Spotify) (3)
- ☐ Get my music for free (4)
- ☐ Other (please specify): (5) ____________________

Q6.8 I use music in my professional life (i.e. music educator, professional musician, music therapist etc.)

- ○ Yes (1)
- ○ No (2)

Q6.9 Please choose the most appropriate category

	Completely Disagree (1)	Strongly Disagree (2)	Disagree (3)	Neither Agree nor Disagree (4)	Agree (5)	Strongly Agree (6)	Completely Agree (7)
I spend a lot of my free time doing music-related activities. (1)	○	○	○	○	○	○	○
I enjoy writing about music, for example on blogs and forums. (2)	○	○	○	○	○	○	○
I'm intrigued by musical styles, I'm not familiar with and want to find out more. (3)	○	○	○	○	○	○	○
I often read or search the internet for things related to music. (4)	○	○	○	○	○	○	○
I don't spend	○	○	○	○	○	○	○

much of my disposable income on music. (5)							
Music is kind of an addiction for me-I couldn't live without it. (6)	○	○	○	○	○	○	○
I keep track of new music that I come across (e.g. new artists or recordings). (7)	○	○	○	○	○	○	○

Q6.10 I have attended _ live music events as an audience member in the past twelve months.

- 0 (1)
- 1 (2)
- 2 (3)
- 3 (4)
- 4-6 (5)
- 7-10 (6)
- 11 or more (7)

Q6.11 I listen attentively to music for_ per day

- 0-15 mins (1)
- 15-30 mins (2)
- 30-60 mins (3)
- 60-90 mins (4)
- 2 hrs (5)
- 2-3 hrs (6)
- 4 hrs or more (7)

Q6.12 Did you skip any tracks (after clicking shuffle) before choosing the one to write about?

- Yes, for the first track I wrote about only (1)
- Yes, for the second track I wrote about only (2)
- Yes, for both tracks I wrote about (3)
- No, not at all (4)

Q70 Did you listen to the track while writing about it?

- Yes, for the first track I wrote about only (1)
- Yes, for the second track I wrote about only (2)
- Yes, for both tracks I wrote about (3)
- No, not at all (4)

Q6.13
Is there anything else you wanted to tell us, for example about the tracks that came on shuffle or your music habits?

____

____

____

____

____

End of Block: Part 3

Start of Block: Debrief/thankyou

Q7.1
Thank you for participating in
Do the Shuffle!

We hope you enjoyed the experience.

Please click the button below to exit the questionnaire.

If you need any more information regarding this questionnaire, please contact the Nordoff Robbins Research Team:
Email: research@nordoff-robbins.org.uk
Tel: 020 7267 4496

If you would like to find out more about the study, please see our website https://www.nordoff-robbins.org.uk/

End of Block: Debrief/thankyou
