# Supplementary material for: Do the shuffle: Exploring reasons for music listening through shuffled play
Source: PLoS One. 2020 Feb 6;15(2):e0228457. doi: 10.1371/journal.pone.0228457 (PMC7004375; doi:10.1371/journal.pone.0228457)
Supplement: S2 File — Finalized code book used by both coders. (PDF) [file pone.0228457.s002.pdf]

**Level 1 Codes**	**Level 2 Codes**	**Level 3 codes**	**Defintion**	**Example**
Association			When the respondent describes or talks about the track by relating it something or someone outside of just the track/music itself	
	Activity		When the respondent describes or talks about the track in relation to its use during an activity. When it accompanies…	
		Accompaniment	... something not covered in the other code or as background music	**"background piece of music"**
		Dancing	… dancing	"Because it is a song myself and my friends like ***to dance to."***
		Driving	… driving	"**Driving too fast**"
		Exercising	… exercising	"So ***I can run to it***"
		Partying	…partying or going out	"***Can party to it*** but also listen to at work, multifunctional"
		Singing	… singing	"This doesn't make anyone else happy as I can't sing in tune, but ***I love to sing this song really loud!***"
		Sleeping	… sleeping	"I enjoy calm solo piano music to either read or ***sleep to***"
		Walking	… walking	"***Strutting down the street***."
		Working	… working,. reading, housework	"Good background music to just put on shuffle while I am busy or ***working***"

	Imagination		When the respondent describes the track though a descriptive narrative or is associated with a season, or time that isn't about a specific memory. When they associate the piece with an image or narrative about …	
		Christmas	…Christmas	"Warm feelings, memories of my youth, **Christmas feeling"**
		Imagination	... something not covered in the other codes, a narrative or story	**"A solitary lonely man playing beautiful music for people"**
		Summer	… summer season	**"Summer** and sunny days"
		Season/Time of Day/Weather	… a time of day, season or weather	"Old movies and **winter"**
	Memory		When the respondent associates the track with ...	
		Event/Concert	... the memory of a specific event, concert, performance or festival.	The track makes me think of **a great night when I saw All Time Low, Good Charlotte and Against the Current in Birmingham** with my brother, the weekend when he visited from uni and before I loved to Lancashire
		Discovery	... the memory or discovering the track for the first time	"***It comes to my mind the first day when I heard the song and decided to save it***"
		Emotion	... the memory of an emotion or feeling.	"***It reminds me*** of being away at school and ***feeling lonely***

				and helpless"
		Gift/Recommenda tion	… a memory of being given it as a gift or as a recommendation not from a specific person	***"It is part of a compilation CD which I was given as a gift***"
		Memory	… general memories	"This song ***brings back good memories*** and the message is a good one."
		Personal Event	... the memory of a specific personal life event.	"**Training at EE**"
		Personal Performance	… the memory of their personal performance or learning of the track	"***I have previously performed this piece (as an orchestral leader and alto singer)***"
		Place	... the memory of a specific place or type of place the music was played.	"It reminds me of some drunken nights ***in our old flat***"
		Rediscovery	… the feeling of rediscovery and remembering the track or the last time they heard it.	"Good song, ***haven't heard it for a while***!"
		Reminisce/Nostal gia	… general nostalgia or reminisce	**"the song make me Reminisce about life"**
		Time or Stage in Life	… a memory of a specific time or stage in their life	"***Reminds me of being young***"
		Vacation	… a memory of a vacation	**"Reminds me of an amazing ski trip"**
	Other Media Forms		When the respondent associates the track with another form of media such as…	
		Film	… a film	"***The film La La Land***"

		Music Video	… a music video	"***I did think of its brilliantly directed and acted music video quite a lot after watching it as it is quite a powerful video***"
		Musical	… a musical	"***I have been to see the musical which it is from (Phantom of the Opera) and so bought the soundtrack***"
		Other Forms of Media	… another other form of media not covered by the other codes	"***The Snap chat filter***"
		Playlist	… a specific playlist	"It's not my favourite ***from the playlist***, but I definitely enjoyed listening to it"
		Television	… a television show	"I grew up in the 80s so it reminds me of when I was first discovering music ***(and MTV)***."
	Person		When the respondent associates the track with a person such as…	
		Being alone	… not being with a person or being alone	"***lonely times"***
		Family	… a family member	"***My daughter*** singing in the car"
		Friends	… a friend	"It brought back funny memories ***with me friends***"
		Loss of a Person	… the loss of a person	"It was a song from a list I made ***to remember my Dad***."
		People	… a group of people	"It reminds me of ***a fun crowd***, sun and fun"

		Recommendation from Family/Friend	… a family member who recommended the track	***"Recommended by family member"***
		Romantic Relationship	… a person they are or were in a romantic relationship with	"***Reminds me of my boyfriend*** when he's away from me"
	Track Identifier		When the respondent associates the track with the informational context around the track like …	
		Album	… the album it is on	"***I have all of The Beatles' albums*** saved because they're obviously amazing."
		Artist	… the artist who created the track	"***Like the artist*** and their music"
		Era	… the era the track is from	"Similar reason - a style and ***era of music*** I like and find relaxing and not too challenging"
		Genre	… the genre of the track	"Yes. I bought this album when I was just becoming familiar with the ***metal genre***"
		Version	… a version of the track	"This song has been on my laptop for a long time because at some point I wanted to listen to more Herman Brood afte***r I had heard his version of "My Way***."
Characteristics			When the respondent describes or talks about the track by characterising it by how it sounds using expressive adjectives or by describing the more technical	

			aspects of the track.	
	Lyrics		When the respondent talks about the track by mentioning or describing ...	
		Lyrics	... the lyrics	"I enjoy the ***lyrics***."
		Lyrical Meaning	... the meaning behind the lyrics	"It has a cool ***meaning behind the lyrics***"
	Musical Expressive		When the respondent describes or talks about the track by characterising it or describing it...	
		Musical Expressive Characteristics	...with descriptive adjectives	"Happy ***bouncy*** eurotrash"
	Musical Technical Aspects		When the respondent talks about the track by mentioning or describing ...	
		Bass line	... the base line	"***It's got a wonderfully syncopated bass line...***"
		Beat	... the beat	"It's on my gym playlist, ***it has a good beat*** and I love the lyrics"
		Hook	... the hook	"It's the best song on the album and has a ***'good hook'***"
		Instrumentation	... the instrumentation	"Lyric is prayerful spiritual conversation and the artists words become my own. ***The musical arrangement is***

				dreamy with cello piano and lead guitar with vibrato. Great tasty drumming"
		Melody	… the melody	"***The melody*** is great"
		Performer	… the performer(s) of the track in references to their abilities or how they sound.	"***Chrissy Constanza's voice*** is exceptional"
		Production	... how it was produced.	"The song does a great job of transporting you into its story as Troye soulfully sings the song's simple yet effective lyrics ***complimented by some excellent production*** which is paced very well feeling subtle at times and then more dramatic as the song goes on."
		Rhythm	... its rhythm.	"Love the story-telling and ***the rhythm***"
		Riffs/Ornamentati on	… the riffs or ornamentations within the track.	"Upbeat, catchy and ***great guitar riffs***"
		Song Structure/ Composition	… its composition or track structure.	**"*Verses are decent, so is the bear during the verses but the chorus is just so boring*"**
		Sound	… its overall sound.	"i like the melody and vocalist ***the way the song sounds*** just makes you feel things you wouldn't feel without it playing"

		Style	… its style.	"It is a very good song, it has a ***very unique vibe/ style***."
		Tempo	... its tempo.	***"High tempo***."
		Vocals	… the vocals within the track, not associated with the performer	"I like how gentle the rhythm and ***vocals*** are"
Evaluations			When the respondent makes an evaluation about the track, an aspect about the track, their relationship, or familiarity about the track.	
	Familiarity		When the respondent makes an evaluation about their relationship with the track by saying…	
		Familiar	... they are familiar with the track.	***"I guess I do, only for the mere fact that I've listened to it quite a lot. I have a relationship based on all the times I've listen to it. Now I know the song perfectly but it has not been always like that, I need listening to it repeatedly to understand it as well as I do"***
		Unfamiliar	... they are unfamiliar with the track.	"It's a fun song but ***I don't know it well***"
	Negative		When the respondent makes a negative evaluation about the track or an aspect of the track.	

		Negative	When the respondent makes a negative evaluation about the track or an aspect of the track.	"No- ***don't like*** arctic monkeys"
	Positive		When the respondent makes a positive evaluation about the track or an aspect of the track by…	
		Favourite	… saying it's their favourite	"***It's my favourite song*** by The Calling"
		Positive	… saying they like it or an aspect of it	"***I like it***"
Responses Induced			When the respondent describes or talks about their response to listening to track, how it makes them feel or what it makes them do.	
	Change		When the respondent describes or talks about their response to listening to track and feeling …	
		Create Atmosphere	…a type of atmosphere	"almost ambient synth intro, **takes its time to create an atmosphere"**
		Calm	…calm	"Also, ***I feel calm when playing this song"***
		Change Mood	… their mood generally change	***"Yes. As I said it can change my mood***, make me feel like dancing and transport me into a different era."
		Energy	…more or less energy	"This song***, boosts up my energy levels*** and gives me the

				mood to work and chill at the same time"
		Happiness/Good Mood	… increase in their mood, positivity	"Yes - t***his song makes me laugh and smile every time I hear it***!"
		Motivation/Focus	… more motivated or focused	"This song was played on the radio where ***the songs motivated me*** to stay active"
		Stress/Frustration /Tension	…less or more stress, frustration or tension	"Makes me think of happy times that lie ahead and helps me to ***escape the stress*** of third year!"
		Sadness	…more or less sadness or crying	**"It makes me feel sad** because of the recent passing of the genius that is Bowie"
	Match		When the respondent describes or talks about their response to listening to track as...	
		Match Mood	... as matching their expectations of what they thought the track would sound like or make them feel or their mood	"It's more upbeat than the last one, and less dirgey, more uplifting, particularly the rising harmonic progression in the middle 8, ***so more suited the mood I'm in which is positive right now***."
