# Supplementary material for: Do the shuffle: Exploring reasons for music listening through shuffled play
Source: PLoS One. 2020 Feb 6;15(2):e0228457. doi: 10.1371/journal.pone.0228457 (PMC7004375; doi:10.1371/journal.pone.0228457)
Supplement: S3 File — Track information given by the participant and track data extracted from “Sort Your Music” application. (PDF) [file pone.0228457.s003.pdf]

Title	Artist	Which App Used	Spotify link	Release	BPM	Energy	Dance	Loud	Valence	Length	Acoustic	Pop.
'Till I Collapse	Eminem	Apple Music	spotify:track:4xkOaSrkexMciUUogZKVTS	5/26/02	171	85	57	-3	10	4:58	8	84
1+1	Beyoncé	Spotify	spotify:track:1pzJboOZaDNwshBnOlNh3a	1/1/11	63	38	30	-7	26	4:34	38	61
101010	Sleeping At Last	Ipad	spotify:track:3uCKzlY6QOHOpxJBsYBYKE	11/8/11	117	38	55	-11	16	3:52	64	31
21 Guns	Green Day	iTunes	spotify:track:64yrDBpcdwEdNY9loyEGbX	5/15/09	160	74	27	-5	42	5:21	5	75
27	Passenger	Spotify	spotify:track:0e7J3sXc7OA6oLiNpF3wUW	1/1/14	178	88	36	-6	66	3:20	2	38
3AM	Matchbox Twenty	Spotify	spotify:track:5vYA1mW9g2Coh1HUFUSmlb	10/1/96	108	67	52	-9	54	3:46	1	67
5 Years Time	Noah And The Whale	Spotify	spotify:track:12je8giC2S9smUHJ81tpiL	1/1/08	122	55	80	-7	75	3:35	40	64
6 Inch	Beyoncé	iPhone iTunes										
7 Ways to Love	Cola Boy	iTunes										
9 Mazurkas in B Major, Op.25: VIII.	Alexander Scriabin	Foobar2000	spotify:track:5UTEMa49K	10/19/93	59	0	22	-36	8	2:37	100	3

Mazurka No.8: Allegretto			KBqHGIZkvg559									
A Ceremony of Carols, Op. 28: V. As dew in Aprille	Benjamin Britten	iPod	spotify:track:2yv77fkF0XWRg8Sg5Cncp9	12/31/95	85	3	22	-26	6	0:59	99	5
A Gleyzele Jas / To the Tavern	London Klezmer Quartet	Iphone	spotify:track:376m9psgJuCDNlpf3UPhvB	9/9/16	112	31	56	-8	81	3:59	78	3
A Little You	Dakota Staton	Ipod	spotify:track:3HkYGca21gcqVcAL4azmSv	3/3/14	122	57	71	-8	93	2:15	63	12
A Nave Vai	Céu	Spotfy	spotify:track:2wz0xgg7iMur9QRe7PyLAh	3/25/16	104	67	82	-8	90	3:48	22	31
A song for the village	E L Heath	Ipod										
A Windmill in Old Amsterdam	Ronnie Hilton	Ipod	spotify:track:7BWMWhcNkr5HVRjRQnxDyk	9/24/07	180	44	54	-12	64	2:51	89	23
About Today	The National	Spotify	spotify:track:0MUh20kYFOG1vN8R6GEAnR	7/20/04	105	60	55	-13	47	4:11	30	59
Ain't Got Far To Go	Jess Glynne	Phone / Spotify	spotify:track:4fXfLQzQaR7cvL8ObPz9Fc	1/1/13	144	84	49	-5	62	3:23	0	53
Al Araby	Syriana	iPhone/Spotify	spotify:track:3nHQy5W7CQwxHzQbY2YpoH	1/1/10	150	41	45	-11	43	5:57	16	8

Alive	Chase & Status	iPhone	spotify:track:74HHgWX80N5YfZpvEQOUov	1/1/13	118	84	33	-4	19	3:26	0	52
All Along the Watchtower	Jimi Hendrix	Laptop/Spotify	spotify:track:2aoo2jlRnM3A0NyLQqMN2f	10/25/68	113	81	44	-6	56	4:01	0	78
All I Want	Kodaline	Laptop - Spotify	spotify:track:2jTidefcsTu6VQhpIImJrt	6/14/13	86	41	21	-10	16	5:06	17	77
All I've Got To Do - Remastered 2009	The Beatles	Samsung smart phone / Samsung Music App	spotify:track:5tztLBvTlNC15Np2tnQ5Ll	11/22/63	119	58	49	-10	88	2:03	22	57
All In The Value	HONNE	Spotify	spotify:track:2vcmVVUN8kd3uiQtlsVr1E	7/13/16	91	63	66	-5	63	3:43	26	55
All That Jazz	Liza Minnelli	Ipod 4	spotify:track:3M3RB8Vw7HaobeuMtfwnPi	12/17/10	137	28	55	-12	76	3:04	66	23
All That Jazz (Originally Performed By Liza Minelli) [Full Vocal Version]	Chart Collective	Ipod 4th generation	spotify:track:6FF7BOOiFdODq7izmEfmfg	9/1/14	135	37	56	-10	65	3:10	25	0
All These Things That I Have Done	Relaxing Piano Covers	Youtube	spotify:track:7o8ziGuASBhKKQgeJK3KTk	1/15/16	219	7	45	-33	5	5:24	100	1
All These Things That I've Done	The Killers	Spotify	spotify:track:0OpSYP9Oly3SqmeIgiRnzT	1/1/04	118	71	55	-7	21	5:02	0	66
Already Dead	Beck	iPad	spotify:track:4DuD1IGLH	1/1/02	115	40	66	-10	34	2:59	17	36

			BEBIjTdquwXkw									
Alright	Darius Rucker	Spotify	spotify:track:6V83U8ls6DZ9obszOawiVR	9/16/08	90	80	60	-5	74	3:52	5	39
Always Be	Jimmy Eat World	Spotify	spotify:track:7LyZKnITD52R7SLxoh2VFq	1/1/07	170	95	39	-4	74	3:05	0	32
American Girl	Tom Petty and the Heartbreakers	iTunes	spotify:track:7MRyJPksH3G2cXHN8UKYzP	11/9/76	115	82	55	-6	78	3:35	45	72
Angel Of Music	Andrew Lloyd Webber	iPhone	spotify:track:5o0xOQhYxr3KqYn4cRE6Ed	12/10/04	78	4	26	-24	26	2:44	96	51
Another Love	Tom Odell	Spotify	spotify:track:3JvKfv6T31zO0ini8iNItO	6/21/13	123	54	45	-9	13	4:04	70	74
Apache	VOVIII	Spotify	spotify:track:3pe4eISKnnOWoJIc9gaa0z	4/20/16	160	81	46	-8	20	3:54	1	54
As Long As I Have A Song (Live)	Beth Hart	Spotify	spotify:track:6x7fzOtzXXN9wF9xM9WKTD	4/13/18	117	17	46	-22	40	3:33	84	30
Astral Plane	Valerie June	Spotify	spotify:track:1ISGSSgVnZhyiOGSI8itNa	3/10/17	173	26	31	-11	21	3:39	80	49
At Last	Eva Cassidy	iPhone/ iTunes	spotify:track:7JskklMjLX2Jhoc5XkwMZE	1/1/00	85	11	52	-12	13	2:59	91	38

Autiosaari	Tuure Kilpeläinen ja Kaihon Karavaani	Spotify	spotify:track:4WxPf0v6WH2UBzTAw5kSOm	10/21/16	90	67	75	-5	85	3:31	9	49
Back In the Saddle	Aerosmith	iPod	spotify:track:0ZGLuduCPjgWY1n85ykgMe	1/1/76	122	90	43	-10	76	4:40	1	54
Bad Bad You, Bad Bad Me	Stephen Fretwell	Ipod	spotify:track:7gUKNmWEXYBJae6k3Zc76C	1/1/04	135	28	51	-12	29	3:26	35	14
Bad Blood	Taylor Swift	iPhone	spotify:track:0TvQLMecTE8utzoNmvXRbK	1/1/14	170	80	65	-6	30	3:32	10	66
Bad Love	White Lies	Samsung phone/Google play music app	spotify:track:0hXs5X3DSmIxe3AZdG2Jxg	1/1/11	148	82	47	-6	52	3:58	0	34
Bad Romance (feat. Caro Emerald)	Caro Emerald	iPad										
Bag Lady	Erykah Badu	iPhone	spotify:track:4FiGJgMiDSLJ90exAXnYdz	10/31/00	76	44	73	-9	56	5:47	35	36
Banana Splits (The Tra La La Song) [as heard in the movie Kick Ass]	The Dickies	Google Music	spotify:track:3m2udfg8tAshZEWqHGjIxA	3/1/10	109	96	44	-5	87	1:42	4	34
Barnes' Yard	The Rural Alberta Advantage	I-phone	spotify:track:1vsTilYEENaqeaIMsP3yJL	3/1/11	153	86	55	-5	72	2:25	0	21
Batancanda	Aproape Unplugged	iTunes playlist										

Be Thou My Vision	Fernando Ortega	I tunes	spotify:track:6T4rV6ClzikL95pSf4NbF1	10/21/98	99	27	61	-12	23	3:53	66	22
Beach - I Wanna Make You Mine	Will Joseph Cook	Iphone - Spotify	spotify:track:0Msm5rX5omqSlhzncBHsTL	4/14/17	168	60	50	-7	56	2:51	0	36
Beautifully Unfinished	Ella Henderson	Phone	spotify:track:0mDsUjzXTMVDLSVw5S8bhi	10/10/14	69	40	45	-9	7	3:38	89	57
Because - Remastered 2009	The Beatles	iTunes	spotify:track:1rxoyGj1QuPoVi8fOft1Kt	9/26/69	81	25	33	-12	16	2:46	75	59
Because Of You	Ne-Yo	Spotify	spotify:track:2VarpyoSpCPNKI56WSAEMN	1/1/07	110	54	81	-6	84	4:27	52	55
Because the Night	Patti Smith	iPhone/Spotify	spotify:track:0lIoY4ZQsdn5QzhraM9o9u	6/15/78	124	76	47	-8	47	3:25	5	62
Been On	G-Eazy	iPhone/Music App	spotify:track:6EXwGsQn4r26YHEjRRpch2	7/18/14	160	66	61	-8	32	3:29	11	40
Behind Blue Eyes	The Who	iPod	spotify:track:0cKk8BKEi7zXbdrYdyqBP5	8/14/71	127	59	39	-8	34	3:41	16	63
Believer	American Authors	Spotify	spotify:track:64ybTt8CKxPdeXBNnu08Op	1/1/14	120	97	58	-3	91	3:04	0	53

Beneath - Remastered	After Forever	Laptop	spotify:track:4QMWMp3O0gPQ0O9IBHfqqT	11/11/16	174	90	42	-7	50	4:52	0	18
Beneath Your Beautiful	Labrinth	Spotify	spotify:track:2EcsgXlxz99UMDSPg5T8RF	3/30/12	84	52	56	-6	23	4:32	23	68
Beta males fairytales	Bend brother	Iphone										
Between The Lines	Bonobo	Spotify app	spotify:track:0oBV4QAfY26kcYqRLRv5ST	10/2/06	99	51	75	-10	49	4:37	57	42
Beverly Hills	Weezer	Laptop/Spotify	spotify:track:1yKu2MhpwzDXXH2tzG6xoa	5/10/05	88	82	69	-4	74	3:16	9	68
Big For Your Boots	Stormzy	Apple Music	spotify:track:3uMB82o0qa7erhiZBW9KQC	2/24/17	175	90	52	-5	63	3:58	3	68
Big in Japan	Guano Apes	Media player	spotify:track:3lQO10djAi0Ym506za76Wt	5/1/00	120	95	48	-7	68	2:48	0	46
Biggest Fan	Brendan Benson	iPod / iTunes	spotify:track:0Bi9Y3VMJ6Bfxl2ZkGlBvm	1/1/05	114	67	45	-6	34	3:43	11	6
Black Bird	Peter Pupping	Spotify	spotify:track:3LCHjekA3EtpXdfyBMeKRa	7/1/07	99	23	44	-15	38	2:14	86	32
Blessed Be the Lord	Joyce Blessing	Spotify	spotify:track:1ck6BECB19dWIQpmM	9/13/14	80	74	62	-4	86	5:03	42	7

			5uCv8									
Blood On Blood	Bon Jovi	iPOD	spotify:track:3e8Tr6rsHLNeoUwYCLZgvy	9/13/88	138	93	36	-6	48	6:17	1	47
Blood on Me	Sampha	Spotify	spotify:track:37el170lJYr5CiWJFk207u	2/3/17	104	52	78	-8	61	4:07	12	60
Blow Your Mind (Mwah)	Dua Lipa	Spotify	spotify:track:7kslwVl5nsh9oefSuuPNN9	6/2/17	109	80	65	-5	49	2:59	2	59
Blue [Da Ba Dee]	Eiffel 65	Spotify	spotify:track:3UCDrOA37BBdcExyIpN3Xj	1/1/99	128	98	78	-4	81	4:44	16	59
Boats Against the Current - Live	Ryan O'Reilly	Default iphone music app	spotify:track:0vn0y8DrfoNa188xik99X5	12/15/14	136	27	52	-12	41	6:17	75	17
Bodyrock	Moby	iTunes	spotify:track:3aTb5EUBOlNDSEcaTFI7Jj	5/17/99	108	87	65	-8	35	3:36	0	49
Bootylicious	Destiny's Child	Desktop	spotify:track:41nT1Sp6ChR65FbsdLlFHW	5/1/01	103	84	84	-4	64	3:28	0	65
Born Slippy (Nuxx)	Underworld	Spotify	spotify:track:1zsDbmrf4ZkjW5hsSsaDjO	1/1/14	140	95	58	-9	30	7:36	0	63
Born to Lose (feat. Gia Koka)	No Sleep	Soundcloud	spotify:track:2oXwrVhXdnQDpYEnr4cAlc	9/12/16	95	54	56	-7	19	3:12	26	42

Boston	Moose Blood	iPhone/Music	spotify:track:4f4pO1KnII2N6plSdq37rh	10/6/14	160	92	40	-6	43	2:32	0	34
Boy Who Cried Wolf	The Style Council	iphone	spotify:track:71EuOv5ncgg5oz9IvTNAtg	1/1/85	98	84	67	-6	84	5:15	20	26
Boys Don't Cry	The Cure	Spotify	spotify:track:1QFh8OH1e78dGd3VyJZCAC	5/11/79	169	84	46	-6	67	2:36	1	69
Braveheart	Neon Jungle	YouTube	spotify:track:0XUvhSzizkEtPgBr1BZ2an	8/26/14	128	73	70	-4	59	3:44	19	53
Break In	CIRRUS	Spotify	spotify:track:5IlwLYrfOVMf2ITB3dEH0o	1/1/97	110	97	70	-8	44	5:27	0	13
Break On Through (To The Other Side)	The Doors	Amazon Music	spotify:track:6ToM0uwxtPKo9CMpbPGYvM	1/4/67	90	75	42	-10	79	2:26	20	71
Breaking The Law	Emeli Sandé	Default iphone music app	spotify:track:6YpwjKx1N6s88hNqgtqo31	1/1/12	118	13	58	-9	33	2:56	87	31
Brighter	Against The Current	Phone	spotify:track:7D8KHKHM0XrmZHu1dloM8H	5/20/16	158	85	46	-4	31	3:27	1	44
Brown Eyed Girl	Van Morrison	Spotify	spotify:track:3yrSvpt2l1xhsV9Em88Pul	9/1/67	151	58	49	-11	91	3:03	18	81
Bubbles	Biffy Clyro	Apple Music	spotify:track:05lBXOMA1uHpVPEQZy	1/18/10	147	72	35	-6	20	5:01	0	54

			joh3									
Bulls In The Bronx	Pierce The Veil	Windows Phone/Groove Music	spotify:track:23UoI3jlFiWdo5jadUYo69	7/17/12	160	93	47	-3	33	4:28	0	61
Burguesinha	Seu Jorge	Iphone	spotify:track:3mXjQTPztvXVRuL6tt6jY3	1/25/08	102	95	68	-5	96	4:18	56	50
Burning Up (Fire)	BTS	Apple Music	spotify:track:4z9gBZQjGS4QLb4LOvmeuA	5/2/16	100	71	63	-3	54	3:23	0	75
Cabinet Battle #2	Christopher Jackson	Spotify	spotify:track:6KRHMYPIWRgFWlXPgqO2Fp	9/25/15	99	54	75	-9	52	2:23	6	63
Cake By The Ocean	DNCE	Laptop on spotify	spotify:track:76hfruVvmfQbw0eYn1nmeC	11/18/16	119	77	77	-5	92	3:39	16	80
Call The Shots	Girls Aloud	iPod touch/Spotify	spotify:track:5uCVGDHnoBBlsVkTlXIWZ9	1/1/07	125	74	64	-5	82	3:45	0	49
Calling You	Jeff Buckley	youtube	spotify:track:0TUFDBFzCh1Hnswo1uQgAH	3/11/16	93	4	35	-17	6	4:59	90	37
Can't Cry	J. Cole	iPhone	spotify:track:212LPeYsfjZjjNbnRhRkbD	7/31/12	73	60	48	-12	37	4:16	16	31
Can't Stop Your Love - Live	Worship Central	Spotify	spotify:track:271asm0EIZHJdxxfW0iO	10/26/14	156	70	34	-7	9	5:54	10	32

			Qt									
Capacity (Breathe)	Jonathan Nelson	Apple music	spotify:track: 0LNoA2DtJeUpMhbscaCbeX	2/1/08	145	29	32	-13	14	4:00	81	18
Capsize	Big Black Delta	Android phone/Spotify	spotify:track: 0oMId7rKJvsQiWiAp1X7fY	4/29/13	98	74	42	-5	19	4:53	0	47
Cash Out	Calvin Harris	Spotify	spotify:track: 0Ws8D3EWUDgY962Xftb0h5	6/30/17	95	79	72	-5	43	3:56	15	65
Castle	Halsey	Deezer	spotify:track: 16w8ZGVSjI4TlTLV8VimBY	8/28/15	130	57	63	-7	16	4:38	25	68
Castle on the Hill	Ed Sheeran	Iphone	spotify:track: 6PCUP3dWmTjcTtXY02oFdT	3/3/17	135	83	46	-5	47	4:21	2	83
Castle on the Hill	Ed Sheeran	iTunes	spotify:track: 6PCUP3dWmTjcTtXY02oFdT	3/3/17	135	83	46	-5	47	4:21	2	83
Castle on the Hill	Ed Sheeran	Spotify	spotify:track: 6PCUP3dWmTjcTtXY02oFdT	3/3/17	135	83	46	-5	47	4:21	2	83
Catch & Release - Deepend Remix	Matt Simons	Spotify	spotify:track: 11BAVEGi1ivJ6JWLqKUNrZ	9/30/15	105	54	84	-9	11	3:15	56	66
Catch Us If You Can	Elle King	Phone	spotify:track: 1QPkehLYh9rHaYtDvzC	4/12/15	122	92	66	-3	69	3:18	2	40

			i3y									
Cathouse	Faster Pussycat	Iphone	spotify:track:4JFum0u7utvjp0aeT6RpHI	7/1/87	167	90	37	-13	48	3:43	7	27
Chaconne	Victoria Mullova	iphone										
Chain of Lakes	Sioux Falls	Spotify	spotify:track:572gFxd0Ahg75t4b5vAHXz	2/19/16	178	64	27	-7	37	2:18	2	15
Chained To The Rhythm	Katy Perry	Itunes	spotify:track:7qvxFz3JodM0A7xEM7k3YD	6/9/17	190	80	45	-5	47	3:58	8	73
Chained To The Rhythm - Oliver Heldens Remix	Katy Perry	Spotify	spotify:track:1j5jfLirlWsytWPm7rmfjk	3/24/17	120	86	72	-5	32	4:37	0	45
Cheap Thrills	Sia	spotify	spotify:track:3S4px9f4lceWdKf0gWciFu	10/21/16	90	70	63	-6	73	3:32	5	75
Cheap Thrills (DJ Metawee Unofficial Remix) - Sia & Sean Paul	DJ Metawee	iPhone music	spotify:track:0iNUvGgr07lUZfbf1Pqyir	1/4/18	100	94	68	-3	92	4:34	29	11
Cheerleader - Felix Jaehn Remix Radio Edit	OMI	Spotify	spotify:track:7vFoFDWqTX0mHzLfrF1Cfy	10/16/15	118	69	78	-6	60	3:01	17	79
Cheerleader - Felix Jaehn Remix Radio Edit	OMI	iTunes	spotify:track:7vFoFDWqTX0mHzLfrF1Cfy	10/16/15	118	69	78	-6	60	3:01	17	79
Cherry	Moose Blood	Spotify	spotify:track:1YOjkOcpU	10/6/14	182	25	37	-13	24	2:40	90	43

			SFs0YBolLpEBt									
Children	Justin Bieber	iPhone Spotify	spotify:track:6O8I0ffpEtyMQmMYo0bj4N	11/13/15	127	89	63	-5	33	3:43	1	58
Chop Suey!	System Of A Down	Spotify	spotify:track:5V3mdRI2yQxbSsJGDPc5lD	1/1/01	127	93	42	-4	30	3:30	0	77
Chwyldro	Gwenno	Android phone/Spotify	spotify:track:2BoIRe1Q3MFA8o2CPqAqcs	7/24/15	120	79	61	-6	50	5:19	32	23
Clair de Lune	Kamasi Washington	Spotify	spotify:track:273VxALm722DRN4PNujOF8	5/11/15	130	35	35	-10	21	11:08	76	37
Clap Track 4	Dorian Concept	Spotify	spotify:track:6TkvfX67Lrwib3l01XaJvN	10/20/14	64	31	24	-10	15	3:18	48	16
Close (In the Style of Nick Jonas & Tove Lo) [Karaoke Version]	Global Karaoke	Laptop on spotify	spotify:track:4vIulJpSe9sfTa2VBDxg1r	5/25/16	124	34	70	-11	16	3:54	66	11
Closer	The Chainsmokers	Spotify	spotify:track:7BKLCZ1jbUBVqRi2FVlTVw	7/29/16	95	52	75	-6	66	4:05	41	87
Cocoon	Milky Chance	iPhone 6	spotify:track:5t2QCWdTPl4UlzkiEbKVCQ	3/17/17	114	71	74	-6	79	4:14	8	52
Com Touch	Clark	Foobar2000	spotify:track:2xKnrarkfG4vP7xx1rs3Mh	4/2/12	90	94	53	-8	34	4:24	20	28

Control (feat. Jay Electronica & Big Sean)	Kendrick Lamar	Spotify/Laptop	spotify:track:4Tbt7DA48fEodJqDTcjXCc	9/15/14	65	93	35	-6	72	7:30	20	41
Correlli	Concerto grosso, D major, Op. 6, Andante	spotify										
Counting Stars	OneRepublic	Iphone	spotify:track:2tpWsVSb9UEmDRxAl1zhX1	1/1/13	122	71	66	-5	48	4:17	7	79
Crazy In Love	Beyoncé	Galaxy S6 / Spotify	spotify:track:5IVuqXILoxVWvWEPm82Jxr	6/24/03	99	77	65	-7	68	3:56	0	78
Crazy Love, Vol. II - Paul Oakenfold Extended Remix	Paul Simon	iTunes Laptop	spotify:track:3EWKerGUrnzI4f1XjikAPP	6/1/18	124	56	81	-8	66	3:55	1	59
Creep	Radiohead	Ipod classic	spotify:track:6b2oQwSGFkzsMtQruIWm2p	2/22/93	92	43	52	-10	10	3:59	1	81
Cricket On The Hearth	Bryan Sutton	Spotify	spotify:track:2mYvNKOmhsBUsWqsXc0Sii	4/29/14	151	71	35	-12	97	2:49	58	27
crushcrushcrush	Paramore	spotify	spotify:track:4m9NfjevXsDVaLtM1kj0Sx	6/11/07	137	79	63	-4	65	3:09	0	63
Cry to Me	Solomon Burke	iTunes	spotify:track:2sCf9tz6LHByczuVT7rqIx	1/1/98	115	62	73	-10	91	2:35	62	60

Dakota	Stereophonics	iTunes	spotify:track:3qaSqrrevBrb84xl5JlptU	1/1/05	147	93	51	-3	31	4:57	13	65
Dapper	Domo Genesis	Laptop/Spotify	spotify:track:2Nh2cMryoXl7BrZoIeN2Pr	3/25/16	113	59	75	-9	56	3:13	7	62
Dasshutsu	Joe Hisaishi	Sansa Clip Sport										
Dazzle	Oh Wonder	Spotify	spotify:track:2KYyhbWmfbW4z8eMhFzme4	9/4/15	112	42	65	-8	61	3:07	59	56
Demons	Imagine Dragons	Spotify	spotify:track:5qaEfEh1AtSdrdrByCP7qR	1/1/12	90	71	50	-3	38	2:58	19	77
Depende	Jarabe De Palo	Mp3	spotify:track:6aaPUBUFw9KEW1p1inVQv9	1/1/98	95	53	70	-12	52	4:25	12	63
Despacito - Remix	Luis Fonsi	Spotify	spotify:track:6rPO02ozF3bM7NnOV4h6s2	4/17/17	178	82	65	-4	82	3:49	23	84
Desperado	Eagles	Spotify	spotify:track:2TjnCxxQRYn56Ye8gkUKiW	1/1/73	60	22	23	-13	18	3:34	95	65
DESPITE THE WEATHER	KAYTRANADA	Laptop/Spotify	spotify:track:5ytmV00jr7di9K0O10dkBO	5/6/16	108	53	78	-9	44	2:02	4	50
Diphylleia grayi	Jonghyun	Phone	spotify:track:5zkaZh7IKPfin3eRNkqfLQ	9/17/15	66	24	34	-10	32	4:35	91	45

Dirty Little Secret	The All-American Rejects	Phone (Samsung Galaxy S6)	spotify:track:2jYmE3Xg0iNDySUm4mqy6Q	1/1/05	144	96	47	-5	45	3:14	0	57
Dog Days Are Over	Florence + The Machine	Spotify	spotify:track:456WNXWhDwYOSf5SpTuqxd	1/1/09	150	81	50	-5	26	4:13	3	70
Dog Days Are Over	Florence + The Machine	iPhone, spotify	spotify:track:456WNXWhDwYOSf5SpTuqxd	1/1/09	150	81	50	-5	26	4:13	3	70
Don't	Ed Sheeran	iPhone	spotify:track:1huvTbEYtgltjQRXzrNKGi	6/21/14	95	61	81	-7	85	3:40	1	77
Don't Know Why	Norah Jones	Spotify	spotify:track:6ybViy2qrO9sIi41EgRJgx	2/26/02	88	20	73	-12	62	3:06	88	73
Don't Let Me Bring You Down	Laura Marling	iTunes	spotify:track:0JqP4OqNrA0fDLYSRjDspO	3/20/15	128	61	48	-10	51	3:10	2	24
Don't Mind	Kent Jones	Spotify	spotify:track:27PmvZoffODNFW2p7ehZTQ	4/25/16	159	77	46	-5	69	3:18	2	70
Don't Stop 'Til You Get Enough - Single Version	Michael Jackson	ITunes	spotify:track:46eu3SBuFCXWsPT39Yg3tJ	8/10/79	119	82	88	-10	95	6:05	13	76
Don't Think	Truckstop Honeymoon	iPod	spotify:track:51QRJyuiwfqADNfZR4N7n1	1/1/08	78	44	81	-12	84	3:31	55	1

Don't Wait	Joey Graceffa	Iphone	spotify:track:5sBdbAZ2a2LJ775CsbkM2h	5/12/15	124	84	68	-7	45	3:26	5	51
Don't Wait	Joey Graceffa	Windows music player on my phone	spotify:track:5sBdbAZ2a2LJ775CsbkM2h	5/12/15	124	84	68	-7	45	3:26	5	51
Don't You Need Somebody (feat. Enrique Iglesias, R. City, Serayah & Shaggy)	RedOne	Phone	spotify:track:1TPUbLdzpfgF7wrPEXIAAB	5/20/16	93	70	69	-6	61	3:28	25	60
Done Wrong	Ani DiFranco	samsung phone music player	spotify:track:23vXAB3EAjSLlLKCGONTUq	5/1/96	120	34	56	-12	8	6:34	7	22
Donny Novitski	Corey Cott	Ipod 4th Generation	spotify:track:59R9kh8emfffKehyQEAxjZ	5/14/18	84	52	56	-8	48	3:45	71	35
dopemang (feat. Ashley All Day)	Kiiara	Spotify	spotify:track:0mnoeoS9UklYuKVbkwCkAX	11/4/16	151	51	66	-6	37	2:49	18	56
Dord Waltz	Elaine "OJ" Wang	Spotify/laptop										
Down In The Tube Station At Midnight - Single Version	The Jam	Google play music on android phone	spotify:track:1EYsaOzRtlTYlfG8BBaJvt	10/14/83	173	81	28	-8	79	4:02	4	44
Down The Line	John Newman	ipod	spotify:track:4AFvk4BAc448o074gE6AuP	1/1/14	142	32	42	-11	23	3:55	81	35

Down Under	Men At Work	Phone/amazon prime music	spotify:track:46RVKt5Edm1zl0rXhPJZxz	1/30/96	107	79	75	-5	89	3:41	5	76
Dr Wu	Steely Dan	Laptop iTunes										
Dream House	Deafheaven	iTunes	spotify:track:6saVnhFqQ2270dJBveTCha	5/28/13	82	80	12	-5	10	9:15	0	48
Drive	Incubus	Apple iphone music app	spotify:track:7nnWIPM5hwE3DaUBkvOIpy	10/26/99	91	79	65	-7	67	3:52	6	77
Du hast	Rammstein	Spotify	spotify:track:5awDvzxWfd53SSrsRZ8pXO	8/22/97	125	92	64	-6	72	3:54	0	70
Dust In Your Pocket	Glass Animals	Google Play Music on an Android phone	spotify:track:714LbDMCzRGIszBfRviwqg	5/28/12	120	38	78	-13	11	4:00	39	48
Dynamite	Taio Cruz	I tunes	spotify:track:2CEgGE6aESpnmtfiZwYlbV	1/1/10	120	78	75	-4	82	3:23	0	75
Dynamite - Acoustic	Sigrid	Spotify	spotify:track:413N9GI94jlsgWzZ4V4mSZ	5/5/17	114	15	55	-11	33	3:52	96	57
Eagle Tax	Mogwai	iTunes Laptop	spotify:track:0lLZnL5JmrUvkbt8wmMmyH	2/25/13	103	47	59	-13	4	3:22	77	26

Early Morning Light	Sarah Jarosz	Spotify	spotify:track:25uT0EtNWSKjICEjTQjZDT	6/17/16	91	4	61	-11	41	2:36	91	41
Easy Street (From the Musical "Annie") [Karaoke Version] - Originally Performed By Original Broadway Cast of "Annie"	A-Type Player	Samsung S6	spotify:track:3iX2bPXAW9U4k0vSiK4gXv	10/4/13	109	26	37	-12	10	4:32	74	0
Emergency	Paramore	Phone	spotify:track:3i4xZSH1kTLE22TUvUFMYo	7/26/05	168	94	42	-4	56	4:00	0	59
Empire (Let Them Sing)	Bring Me The Horizon	iPod	spotify:track:1DbdQp5dwuawBGmxSHw6Fr	3/29/13	97	96	44	-3	26	3:46	0	52
En hjältes väg	Raubtier	Computer	spotify:track:1yWN5e8qLIx9zluOJnS8q4	9/22/10	150	95	42	-4	33	4:42	0	39
English Rose	Ed Sheeran	Spotify	spotify:track:3B8BedGdUGT1Ixog6v8ua8	11/13/15	115	65	58	-7	18	3:04	10	55
Epidemic	Slayer	iTunes	spotify:track:3wDpbLxFuMEYN0QcC2z393	1/1/86	98	100	40	-4	9	2:23	0	34
Étude No. 3 in E Major, Op. 10: III. Tristesse	Frédéric Chopin	Spotify	spotify:track:6FoU9AHorRU5EsBGFNbssf	8/29/17	72	6	40	-23	4	4:12	100	36
Everybody Knows	Leonard Cohen	iPod	spotify:track:60s0QWaOZ2UTzqdIHB	2/2/88	104	46	79	-14	69	5:35	64	55

			Ct3x									
Everything	Michael Bublé	iPod	spotify:track:4T6HLdP6OcAtqC6tGnQelG	4/27/07	123	69	69	-5	49	3:32	39	72
Everything Your Heart Desires	Daryl Hall & John Oates	iPod classic	spotify:track:4AiVt49hSjkkArFOIlAZhL	7/27/88	100	86	71	-6	89	5:00	16	39
Everywhere You Go (Villalobos Celestial Voice Amnesia Resurrection Mix)	Mari Kvien Brunvoll	Spotify	spotify:track:4SQnDqH8UfgciQ9GTL0MXY	3/24/14	120	36	81	-16	41	28:32:00	0	39
Ex Calling	6LACK	Spotify	spotify:track:1MQ8ydvrTnn2QJgXFATJvD	11/18/16	156	56	77	-7	6	3:32	1	75
Exiles	David Cross	iPod	spotify:track:2M8G0wzsuM1gwwAVVRDY8I	4/1/10	82	46	50	-12	38	5:59	7	13
Experience The Jewel	Wintersleep	iTunes										
Eye Of The Beholder	Metallica	iTunes	spotify:track:7p2yIOf8YXJUSmUnG3TAWr	8/25/88	122	81	64	-9	59	6:30	0	51
Facedown	The 1975	spotify	spotify:track:66mhOWjoTLTXm7y7V1eHRs	9/2/13	141	43	14	-12	3	2:49	11	42
Faded	Alan Walker	Spotify	spotify:track:7gHs73wELdeycvS48JfIos	12/4/15	90	65	59	-5	17	3:33	3	84

Faint	Linkin Park	iTunes	spotify:track:7AB0cUXnzuSlAnyHOqmrZr	3/24/03	135	98	55	-4	59	2:43	11	66
Fake Plastic Trees	Radiohead	iTunes on iPhone (also used to complete this survey)	spotify:track:045sp2JToyTaaKyXkGejPy	3/28/95	74	23	45	-13	13	4:51	17	69
Falling Slowly	Glen Hansard & Marketa Irglova	Spotify	spotify:track:7M3FoO2rvMsAeRTCg1hYoe	8/21/06	138	33	28	-9	11	4:53	54	29
False Hope	Laura Marling	Ipod	spotify:track:3CIvt8YmxXMUE5mCOQ7Cz9	3/20/15	134	72	46	-9	36	3:12	2	34
Fanfare for the Common Man - Single Edit	Emerson, Lake & Palmer	Spotify	spotify:track:0dkL8m14x0wecbq04QfBKs	1/1/94	73	44	37	-14	27	2:59	41	46
Fans	Kings of Leon	Apple Music on iPhone	spotify:track:6LEJsHo21kCF3Bf1kpSN76	4/3/07	104	72	42	-6	35	3:36	3	56
Fifty On Our Foreheads	White Lies	Spotify on my PC again	spotify:track:7MuM8ChFTheRVvVnyI8p4m	1/1/09	112	68	58	-7	53	4:22	0	31
Fight The Power	Public Enemy	iPhone - Spotify	spotify:track:26zWdOUpB6P9E9OtQQc0qp	4/10/90	106	59	80	-13	40	4:43	0	50
Find My Way / Finale	Sheridan Smith	Amazon Music on mobile	spotify:track:69TuvlfXJ9UB7VwmhBetgU	8/16/10	117	41	47	-8	38	4:26	42	20

Fineshrine	Purity Ring	sound cloud	spotify:track:5KeyVNymqfqac1wLDseK8v	7/23/12	130	80	61	-4	40	3:30	2	60
Fireball	Pitbull	Apple iphone	spotify:track:49eplIHvgCLY8HAgcaWrUb	7/23/14	123	94	69	-5	79	3:55	9	68
Firestarter	Fedde Le Grand	Spotify	spotify:track:4OA689P6GbokYYksWgyKKm	7/28/17	107	94	71	-5	72	3:17	5	40
Firework	Katy Perry	Spotify	spotify:track:4lCv7b86sLynZbXhfScfm2	3/12/12	124	83	64	-5	65	3:48	14	73
Five Hebrew Love Songs: Kalá Kallá (Light Bride)	Eric Whitacre	Ipad	spotify:track:1nxLqMNg5Cj4FlNJm56jQw	1/1/10	134	8	26	-23	8	3:08	95	24
Flash, Bang, Wallop	Tommy Steele	Ipod 4	spotify:track:0KBnDlBPw4W89jBt6bmCg5	1/1/05	122	89	74	-6	84	2:19	57	25
Flaws	Bastille	Apple Music	spotify:track:0aUr2ZD1NEGaPA4lbGxI2I	1/1/13	144	66	57	-7	41	3:39	41	54
Focus	Ariana Grande	Spotify	spotify:track:1cdzfFjEbUbgTm5nv3FgXR	10/30/15	100	88	68	-6	82	3:31	27	66
Fool	BØRNS	Spotify	spotify:track:1huRm0I1QxVb1pJziyCSRh	10/16/15	125	80	68	-5	89	3:38	0	53

For Those Below - Bonus Track	Mumford & Sons	Spotify	spotify:track:0yWexgCswlhweroA1bnPz3	9/21/12	180	45	26	-9	38	3:36	64	34
For What It's Worth	Placebo	Play Music	spotify:track:3R7UvtRUySTlEBMTllk9Ee	6/9/09	142	76	57	-8	55	2:47	0	45
For Whom The Bell Tolls	J. Cole	Spotify	spotify:track:3MWlVSkoLS1e66nlZ2tuWJ	12/9/16	112	41	65	-11	17	2:08	47	63
Fresh Feeling	Eels	iPhone/Spotify	spotify:track:5zIMD8C7t1qiIXThhgDIpD	1/1/01	94	73	76	-8	51	3:37	5	43
Frim Fram Sauce	Diana Krall	IPhone	spotify:track:4KQDdFvLo9qAo3BbMenJ25	1/1/07	117	28	76	-12	42	5:00	93	37
Frosty The Snowman	The Jackson 5	iPod Classic	spotify:track:5B6xtd8cW58ZqAO9Z3bBUj	10/15/70	120	62	67	-8	94	2:39	46	21
Games People Play	The Alan Parsons Project	Phone	spotify:track:2gQsUHVFY4tpXc3AFU2mcS	11/1/80	132	68	72	-11	69	4:23	21	53
Get Out	Shlohmo	Spotify	spotify:track:0liowmKvYbRnWqGN8Zybl4	8/9/11	111	50	51	-5	4	4:37	82	27
Getting Late	Floetry	iPhone	spotify:track:0khnsS3vcqT6qngEhZCk4c	1/1/02	154	30	49	-9	40	6:47	26	49

Girl of the north country (live)	Bob Dylan and Johnny Cash	Youtube										
Girls In Their Summer Clothes	Bruce Springsteen	Spotify	spotify:track:5kVSDFEAqgjKtcD6tRvRzY	9/25/07	113	76	44	-4	67	4:19	3	45
Give It to Me (Karaoke Version) - Originally Performed By Timberland Ft Nelly Furtado and Justin Timberlake	Sing Karaoke Sing	Spotify	spotify:track:2oyNPKqBLezdiNyaXEQFvQ	10/5/12	111	56	92	-11	60	3:56	12	5
Glass and Buffalo Warrior Travel	Ryuichi Sakamoto	Spotify	spotify:track:2yG7nhSqBuMhBj3I2a5k9S	1/7/16	126	11	17	-35	4	1:51	97	29
God Rests In Reason [Live]	Jason Mraz	Spotify	spotify:track:0PSpPABGFLN4D9Iqa46CQj	2/13/07	105	28	33	-12	15	4:51	81	26
Goldberg Variations, BWV 988: Aria da capo - 1981 Version	Johann Sebastian Bach	Desktop computer/Spotify	spotify:track:1aTpzY2ggWHhPi8p2f56u1	8/27/02	135	1	44	-37	28	3:44	100	38
Golden Skans	Klaxons	Spotify	spotify:track:6BqWhxll86CGGE6WxgdRqG	1/1/07	142	84	47	-3	77	2:45	0	65
Gone, Gone, Gone	Phillip Phillips	I Music	spotify:track:56sxN1yKg1dgOZXBcAHkJG	1/1/12	118	66	67	-6	50	3:30	14	47
Goodbye	Miley Cyrus	iPhone music	spotify:track:5ddXNYCr4kbUXzRpvqA58W	1/1/08	175	70	36	-5	20	3:51	1	29

Gravel	Ani DiFranco	samsung phone music player	spotify:track:7LjFcq4C2NGlXrSadUma79	2/1/98	139	58	76	-9	86	3:32	31	32
Great Ideas	Fake Laugh	Spotify	spotify:track:7f0fX8mDWKHmZorgOMqd2Q	10/7/16	150	77	57	-8	93	3:01	1	18
Grown-Up Christmas List	Amy Grant	iPhone	spotify:track:24lCPIiEWKg9K7k1Gh1h8q	1/1/92	122	20	32	-9	14	5:02	85	22
Gypsy	Fleetwood Mac	Spotify	spotify:track:19Ym5Sg0YyOCa6ao21bdoG	11/21/88	131	54	62	-14	85	4:25	2	69
Hallelujah	Jeff Buckley	iPhone, Spotify.	spotify:track:74X1epeRufHckhuX1KFD04	1/1/94	101	14	34	-11	9	6:53	92	72
Hand Covers Bruise	Trent Reznor and Atticus Ross	Shuffeld Youtube playlist	spotify:track:1v0uVPU6BWcbog5BiWLWVa	9/28/10	115	15	21	-25	4	4:19	97	48
Hanging On The Telephone	Blondie	Android phone/Spotify	spotify:track:0e17OFidbbC7GYP2KFxhct	10/31/81	151	77	50	-12	55	2:33	2	43
Happy	Pharrell Williams	iPod	spotify:track:6NPVjNh8Jhru9xOmyQigds	6/18/13	160	76	65	-7	96	3:53	29	75
Happy Little Pill (Casper Zazz Remix)	Troye Sivan	sound cloud										
Hard To Love	Old Crow Medicine Show	iPod	spotify:track:2yd6XoFVvbs4x8hMTVR	2/10/04	151	74	57	-6	80	2:31	63	34

			OIx									
Havana Gang Brawl	The Zutons	Spotify/laptop	spotify:track:2rPTTO4BvAs9P8crblrpEa	1/1/04	160	89	49	-6	45	4:30	8	26
He Like That	Fifth Harmony	Spotify	spotify:track:1zjcIIrm2fbVFmG3dsj03e	10/29/17	147	68	59	-6	49	3:37	36	69
He of the Name	Revo	Cell phone										
Heart of the City	Guthrie Govan	Spotify	spotify:track:0pcwaoBxngoFE5qIL8FDWy	5/13/13	81	46	40	-7	35	2:00	1	38
Heart Skips A Beat	Olly Murs featuring Rizzle Kicks	Galaxy S6 / Spotify										
Heartbeat	Scouting For Girls	iPod nano	spotify:track:20Mf1Payc1BqR82y5zEvl0	9/14/07	155	86	56	-4	86	2:56	5	57
Heartbeat Song	Kelly Clarkson	Play Music	spotify:track:4WGgAdWVeF0ExmWow9R9XY	3/4/16	149	80	49	-4	49	3:19	1	53
Heartbeats	José González	Spotify/Phone	spotify:track:3jQyadhLTxpxadQlkFh2b8	1/1/03	178	15	39	-17	31	2:40	98	65
Heaven Knows	The Pretty Reckless	YouTube	spotify:track:0ifIudDeWelhhKzNqP12jm	3/17/14	84	80	54	-4	58	3:45	8	57
Heaven's Knife	Josh Garrels	Apple Music	spotify:track:0jLWlbEJYg	4/7/15	183	26	25	-9	22	3:23	81	48

			WeKnzHhvKUbz									
Hello	Beyoncé	iPhone iTunes	spotify:track:3ID5lLzkh4uM8XPUuB2VKq	11/14/08	98	77	33	-6	42	4:17	21	50
Hello, Dolly!	Louis Armstrong	iMusic	spotify:track:1ahQsOwEQMuqh7DgUvYSGN	1/1/64	155	41	58	-10	62	2:27	84	46
Hello, Goodbye - Remastered 2009	The Beatles	iPhone	spotify:track:0vZ97gHhemKm6c64hTfJNA	11/27/67	99	73	48	-9	81	3:29	31	64
Helplessly Hoping - Remastered	Crosby, Stills & Nash	Spotify	spotify:track:1UKobFsdqNXQb8OthimCKe	5/29/69	147	16	57	-14	44	2:38	91	67
Hero	Family of the Year	Spotify	spotify:track:6GRDI9suQHikFP6euIXnpq	7/10/12	175	54	34	-6	27	3:10	21	66
High And Low	Empire of the Sun	Spotify	spotify:track:34WI9hCaE3AzbOYT9wFXLP	10/28/16	127	77	51	-5	32	3:45	2	60
Higher	Taio Cruz	iPod touch/Spotify	spotify:track:3IzaEzfHPfxG3uolyTARny	1/1/10	128	91	53	-4	54	3:08	3	45
Hips Don't Lie	Shakira	Spotify	spotify:track:3ZFTkvIE7kyPt6Nu3PEa7V	3/27/06	100	82	78	-6	76	3:38	28	84
Hit the Road Jack	Ray Charles	iPhone-iTunes music	spotify:track:49fzPkBb3aOUWYRKa	2/23/12	173	62	61	-12	94	1:58	57	68

			TWVhm									
Ho Hey	The Lumineers	Spotify	spotify:track:1jdNcAD8Ir58RlsdGjJJdx	1/1/12	80	47	69	-9	36	2:41	78	72
Hold My Cock, Living on a Prayer Parody Bon Jovi Joke	Funny Explicit Comedy Tunes	IPOD	spotify:track:5cn2iaghfMeWCjWvovI7JC	5/28/11	123	58	64	-11	53	4:01	32	16
Hole In My Heart	Alphabeat	iPod touch	spotify:track:4Gnb5G5K53X7wETeaJamka	1/1/10	130	88	66	-3	73	4:09	16	24
Holiday/Boulevard Of Broken Dreams	Green Day	Samsung music	spotify:track:0MsrWnxQZxPAcov7c74sSo	9/21/04	167	83	29	-3	45	8:13	0	74
Holy Ground	Taylor Swift	Apple Music	spotify:track:3KmYVOuK2eG7LGP7qQ8DLR	10/22/12	157	82	63	-7	65	3:23	2	48
Honey Sweet	Blossoms	Spotify	spotify:track:4D2rEIyYw0meeh8AOXqfxK	8/5/16	110	71	57	-6	63	3:36	3	59
Honey Sweet	Blossoms	Spotify	spotify:track:4D2rEIyYw0meeh8AOXqfxK	8/5/16	110	71	57	-6	63	3:36	3	59
Hoover Factory	Elvis Costello	Spotify	spotify:track:1mqOWiSlqZcXHRrC2Q0Jdx	1/1/80	134	29	53	-18	76	1:44	76	15
Hot Love - A Side	T. Rex	iPhone-Apple Music	spotify:track:1FvG5AZm68WSFQj3fJy	9/24/71	131	54	56	-9	49	4:55	8	52

			woy									
How Beautiful (Live)	Mosaic MSC	Spotify	spotify:track:0J9TFCYyjLHYJOcenwxblB	9/16/16	159	41	22	-8	8	6:56	51	56
How To Disappear Completely	Radiohead	Spotify	spotify:track:69pwmeyvQMuHMtkCmpEWhQ	10/1/00	102	30	17	-12	21	5:56	32	58
How to Save a Life	The Fray	Spotify	spotify:track:5fVZC9GiM4e8vu99W0Xf6J	9/13/05	122	74	64	-4	36	4:23	27	81
human	Christina Perri	Play Music	spotify:track:1zVhMuH7agsRe6XkljIY4U	11/18/13	144	49	44	-6	25	4:11	13	71
Human Fly	The Cramps	Android phone/Spotify	spotify:track:0ElRzK07sc9eszyk1ea9Ab	1/1/89	120	85	53	-11	31	2:16	4	53
Humming	James Martin	iTunes										
Hummingbird	B.B. King	Itunes on Ipod	spotify:track:0CF1X2oxbHWyFNPG2CPeNX	1/1/05	162	59	53	-9	65	4:41	53	51
Hundred Mile High City	Ocean Colour Scene	Iphone	spotify:track:0EgOQEj1fHUyoPAkkp03Hp	1/1/97	112	94	42	-4	70	3:58	0	47
Hyena	the GazettE	spotify	spotify:track:7pKVcmlJSTDNMt3QOYrGzH	7/4/07	125	98	50	-4	41	4:16	0	24

I Believe In You - 1997 Remastered Version	Talk Talk	Spotify	spotify:track:3SzoVeS9Eaxt6dmfXF0fPo	1/1/88	91	19	52	-22	16	6:10	69	38
I Could Die For You	Red Hot Chili Peppers	Laptop iTunes	spotify:track:3oP2vxUR2bjFCrU9YolGnM	7/9/02	170	73	38	-4	39	3:13	10	54
I Feel A Change Coming On	Michel Montecrossa	Ipod	spotify:track:3G1sfbV448J36wqmpEITbt	2/20/10	140	64	42	-7	39	5:36	18	0
I Know A Place	MUNA	Iphone / Spotify	spotify:track:0bPSRn4crnh5f1JhELPlyL	2/3/17	112	90	69	-5	78	4:33	14	57
I Like The View	Lil Wayne	Sony	spotify:track:6Arm9x83wtStSfxAQ3pnjJ	1/1/11	96	85	52	-6	39	4:41	0	39
I Love It Loud (Broadcast 1988)	KISS	iPhone	spotify:track:7yDrKl1J4VWNbR4aLU3qJ7									
I See My Mother	POLIÇA	Apple Music / Iphone	spotify:track:4Q8c40BEovg8KXHfo28rhn	4/30/12	130	72	66	-9	26	4:26	1	25
I Surrender	Rainbow	Spotify	spotify:track:0gGJO7JM51uAUu7EE2lxx6	1/1/81	133	86	50	-5	78	4:02	16	54
I Wanna Feel - Radio Edit	Secondcity	Spotify	spotify:track:77jaOS5YDCDGPto5Pomi4h	5/23/14	122	90	69	-6	80	3:06	8	61

I Wasn't Worried	Julia Nunes	iPhone 5S (Music app)	spotify:track:6i3t5gEzbPw2Gt8aTGqrQV	2/28/12	78	14	34	-12	11	0:57	96	16
I Will Always Love You	Whitney Houston	Spotify	spotify:track:4eHbdreAnSOrDDsFfc4Fpm	11/17/92	67	21	31	-13	11	4:31	84	75
I Will Remember	Toto	AGPTEK MP3 Player	spotify:track:1MzFmVDSl7Vm00f3Hw4Xfx	5/1/95	98	51	65	-9	45	6:06	15	53
I Won't Back Down	Tom Petty	Spotify	spotify:track:0Ir0Esfpcg0EB6Kq8VbbAh	1/1/89	114	47	77	-14	96	2:56	4	62
I Would Like	Zara Larsson	Itunes	spotify:track:0k9gqa8mjVMeoFuGIsudtY	3/17/17	121	71	49	-4	30	3:47	9	57
I'll Be There	The Parlotones	iPod	spotify:track:2ZgRR5h16TOLXFLYqXgJ8e	6/1/05	184	58	33	-7	25	3:39	48	26
I'm a Little Teapot	Nursery Rhyme Street	iphone	spotify:track:00F4r5l7tBBugtSq4AVRMJ	1/22/16	199	7	64	-16	79	0:43	80	11
I'm Good - Radio Version	Blaque	iPod Touch	spotify:track:7EgG37pjxw0Y4GHwjK3CBS	9/2/03	103	80	66	-5	71	3:36	3	48
I'm Not Ready	Yeek	Soundcloud	spotify:track:6z3fmpMqgV3a8DYZgdiU7w	12/10/15	120	72	80	-5	56	4:06	4	49

I'm Not That Girl - From "Wicked" Original Broadway Cast Recording/2003	Idina Menzel	Spotify	spotify:track:6J2ZT1S0t9kMGQQJUIKQJz	1/1/04	77	7	25	-19	20	2:59	95	48
Identity	Ska Cubano	ipod classic										
If I Can Dream	Elvis Presley	Spotify	spotify:track:5UR5aIbcfEdivfsPin8G45	10/30/15	188	71	17	-7	34	3:12	24	42
Ignition - Remix	R. Kelly	i tunes	spotify:track:5dNfHmqgr128gMY2tc5CeJ	1/1/03	133	52	80	-7	86	3:06	6	75
Il Volo	Relajante X	Spotify	spotify:track:2oCyRkpbb38088y39rEZNZ	9/21/16	87	5	15	-24	4	2:34	91	13
Imagine - Remastered 2010	John Lennon	iTunes	spotify:track:7pKfPomDEeI4TPT6EOYjn9	9/9/71	76	26	55	-12	17	3:08	91	76
In My Head	Jason Derulo	Spotify	spotify:track:6St9lR6dhV2tpCNVz7qfrS	2/26/10	110	75	76	-4	85	3:19	3	70
In the End	Passenger	I pad	spotify:track:6dO5oQiCCvciPZOlUNEmb6	7/28/17	83	12	60	-17	26	3:05	77	48
Inside the suitcase	From Magical Beasts and Where to Find Them	iPhone itunes										
Interested in Music	Adam Green	Phone, Amazon Music	spotify:track:3jeUy1caCBqmZ7pcR1r	4/29/16	94	79	71	-7	91	1:16	7	16

			GVp									
Into The Wild	LP	Spotify	spotify:track:2gY0Ff8dmJNlCyQxDDqBEf	8/25/17	144	92	55	-4	24	3:55	4	55
Intoxicated - Radio Edit	Martin Solveig	Spotify	spotify:track:5ZKqR1beprJ3wa6wGUwTWH	5/18/15	125	68	81	-4	47	2:41	1	63
Introduction and Variations on Trockne Blumen from Die schone Mullerin, Op. 160, D. 802: Variation 4	Franz Schubert	ITunes on laptop	spotify:track:6bfxK8C4W855X25sgKpD6i	3/31/09	95	8	33	-20	27	1:47	99	4
Introduzione (Andante non troppo -- Allegro vivace) from Bartok's Concerto for Orchestra	erbert von Karajan (...and an orchestra! I can't remember anymore and it doesn't say...Berlin Phil perhaps?)	iPod										
Invisible Touch - Remastered 2007	Genesis	Spotify	spotify:track:480dDrqG7LO6qDaphHeXlM	6/9/86	131	94	62	-6	84	3:30	20	63
Issues	Julia Michaels	iPhone 6	spotify:track:0xG0SHshCStMEyNbNC5d4k	7/28/17	114	42	69	-7	45	2:56	39	75
It Ain't Necessarily So	Brian Wilson	default player on Lg Android phone	spotify:track:7v4184qZkpjKG7blTGOGxj	1/1/10	84	43	50	-9	51	3:58	48	4

It Could Happen To You	Miles Davis Quintet	spotify	spotify:track:34xixFi5y3I5FIOH1ZWisx	1/1/58	94	40	62	-11	63	6:37	45	32
It Just Won't Do - Radio Edit	Tim Deluxe	iPhone	spotify:track:6Ss1QOGzn0iG8hrRRSGrr9	11/28/11	130	94	68	-5	88	3:18	0	53
It Was A Very Good Year (with Willie Nelson)	Ray Charles	iPod	spotify:track:3klDKws1sU7Th1htqC5iDO	8/30/04	94	18	23	-12	6	5:00	85	26
It's A Vibe	2 Chainz	Spotify	spotify:track:6H0AwSQ20mo62jGlPGB8S6	6/16/17	73	51	82	-7	52	3:30	3	79
It's Teavee Time	Charlie and the Chocolate Factory the Musical	Iphone Music										
Jingle Bells	Diana Krall	Iphone music app	spotify:track:6Kr6DLRJIA1SeOo9EI9t0G	1/1/05	101	55	71	-6	58	3:26	37	22
Joy - Remix	Rend Collective	Mobiel Phone (Vodafone Smart Ultra 6) usign Play Music app	spotify:track:3r1xRl865rc7h4C6DmUPpL	3/17/14	128	82	57	-6	62	4:26	0	18
Just Hold on (Originally by Steve Aoki and Louis Tomlinson) - Instrumental Version	Karaoke Freaks	Spotify	spotify:track:1CIehFaI6h75Lg74pXiyjW	12/13/16	116	58	65	-12	13	3:14	0	11

Just Hold on (Originally Performed By Louis Tomlinson & Steve Aoki)	Sunfly Karaoke	Spotify	spotify:track:3XxfyVPlCzwFvrqcmGnXmb	1/25/17	115	74	70	-6	38	3:19	0	0
Just Looking	Stereophonics	Sonos	spotify:track:1vltPOVBSb4Ib1JNVAfTYv	1/1/99	100	49	34	-6	4	4:13	13	48
Kaleidoscope	Urban Rescue	Spotify	spotify:track:75dL1cqk3oqV1QP3NNaOdv	5/6/16	110	95	52	-7	36	3:57	0	39
Keep On	Kehlani	Apple music	spotify:track:4MV4wa3Dke1GMBtk06GMR0	1/27/17	98	45	74	-8	51	3:29	26	69
Keep You in Mind - Remix	Guordan Banks	iPhone/Music	spotify:track:5uKryGXlPNcs1hqq7nCbVe	11/2/16	168	66	53	-6	38	4:26	41	49
Keeper	With Confidence	Itunes	spotify:track:7C1aLb3VN8Y5r3btsMBkwB	6/17/16	197	94	41	-4	61	2:41	0	52
Keyboard Sonatina in D Minor, HWV 581	George Frideric Handel	Windows media player	spotify:track:0RS5oWPJ23MuDgo0bGC8KK	1/1/16	82	78	31	-10	96	1:38	92	3
Killing Me Softly with His Song	Fugees	Iphone	spotify:track:1MAqR81Tz28IIqMJ2KUDAO	1/1/96	92	29	77	-17	51	4:59	3	73
King & Country	Seth Lakeman	ITunes	spotify:track:6bJC82TCv4CF2b7o4k1TGc	8/15/06	145	44	64	-8	52	4:27	82	23

King Of The Mountain	Kate Bush	Google Play Music on Android phone	spotify:track:7u62wiKKB55AAbHjJa5RPV	10/3/05	150	50	58	-12	61	4:54	38	36
Kinky Afro	Happy Mondays	Spofity	spotify:track:2ItPID4dgSL1t1ZbZCrg5S	1/1/99	118	76	55	-13	72	3:59	0	55
Kitchen Sink - Bonus Track	Twenty One Pilots	Play Music	spotify:track:0SUiF6BG2OPfqorlag9vlM	1/4/13	120	59	65	-8	51	5:34	46	49
L-O-V-E - Long Version	Joss Stone	spotify	spotify:track:4MymCsjbOkhuP2kWNSZgLo	1/1/11	95	78	53	-6	65	2:49	38	54
La mer	Charles Trenet	Spotify	spotify:track:7sZSe99XSTu5KpYJEThvWr	6/25/90	69	17	22	-16	55	3:22	97	57
La sonnambula: Oh! Se una volta sola rivederlo io potessi - Ah! Non credea mirarti - Ah! Non giunge uman pensiero - From La Sonnambula	Vincenzo Bellini	Ipod	spotify:track:0ilzA0mO7aYterGE9xMXZB	6/10/11	70	10	23	-20	4	14:12	98	1
La Vida Es Limonada [Radio Mix]	Marquess	Spotify	spotify:track:4t6g5Dd52NljBIwzlsvdp1	6/20/08	120	90	64	-6	92	3:29	6	26
Laid Back	Big Fin	iTunes										
Lambada - Original Version 1989	Kaoma	Spotify	spotify:track:6xepovPqjvrkEw9Y5AMmTm	11/13/07	119	80	76	-5	97	3:27	21	64

Land of Nod - Lack of Afro Mix	The New Mastersounds	iTunes	spotify:track:1k1PdCcY8XA58B8yyZMLpC	11/12/07	108	90	75	-7	42	3:50	11	10
Landslide	Fleetwood Mac	Computer/Spotify	spotify:track:5ihS6UUlyQAfmp48eSkxuQ	7/11/75	159	16	41	-22	42	3:19	88	75
Lansana's Priestess	Donald Byrd	Spotify app	spotify:track:6e0UKhWk7vZFPEgRLVUNDg	1/1/73	117	88	48	-9	89	7:39	26	47
Larger Than Life	Backstreet Boys	iPod Classic	spotify:track:6sbXGUn9V9ZaLwLdOfpKRE	5/18/99	108	97	66	-3	75	3:53	11	66
Laura Palmer	Bastille	iPhone 6	spotify:track:0VszWViF3bhfCvxhDVC3aM	1/1/13	135	79	57	-5	37	3:06	2	50
Lay Me Down	Sam Smith	iPad	spotify:track:64GRDrL1efgXclrhVCeuA0	11/6/15	125	19	47	-11	33	3:40	92	77
Learning To Fly	Tom Petty and the Heartbreakers	IPhone	spotify:track:17S4XrLvF5jlGvGCJHgF51	1/1/91	117	77	65	-10	95	4:02	21	66
Legacy Of Tudors	Serenity	Spotify	spotify:track:4asjRo9SsNqVWMJ8vysBpC	8/1/13	160	94	46	-2	41	5:02	0	4
Legend Has It	Run The Jewels	Spotify	spotify:track:6bGwloiyyHXwFZ4yV1zjqR	12/26/16	121	56	83	-7	47	3:26	3	58

LEMONADE	SOPHIE	Spotify	spotify:track:4rcxSQEuGn8ZXvFtbcNDGZ	11/27/15	71	65	83	-5	84	1:59	30	45
Let Everything That Has Breath	Matt Redman	iTunes	spotify:track:3eFti0TnNRLlfgiWu0WoXo	4/25/10	118	90	49	-6	28	4:26	0	23
Let It Be - Remastered 2009	The Beatles	iMusic	spotify:track:7iN1s7xHE4ifF5povM6A48	5/8/70	143	40	44	-8	41	4:03	63	76
Let It Go	James Bay	Spotify	spotify:track:40EB7ABUO6MoWMUwPKptJ7	3/23/15	147	31	55	-10	25	4:21	82	69
Let Me Love You - Tropkillaz & Mc Livinho Remix	DJ Snake	Spotify	spotify:track:6hLyfWFe42YtHONpJZDt4p	12/19/16	100	87	66	-5	33	3:36	6	42
Let's Work	Prince	ipod classic	spotify:track:0wgVCOPXv9YSgmRijmDSkh	10/14/81	120	44	82	-15	90	3:55	1	36
Let's Work Together - Long Version / Bonus Track	Canned Heat	iTunes	spotify:track:7vJaNv4ItDyYXXCVRJreh6	8/3/70	119	71	75	-12	87	3:14	12	49
Life Is Just A Bowl Of Cherries	Johnny Mathis	Spotify	spotify:track:5NVrv0B0XtMLxXYoX9toww	1/1/04	105	41	52	-10	32	2:45	84	10
Life On Mars? - 2015 Remastered Version	David Bowie	Spotify	spotify:track:3ZE3wv8V3w2T2f7nOCjV0N	12/17/71	123	38	44	-15	22	3:56	64	71

LIFTED	CL	Spotify	spotify:track:2DHWM8Bm4zuzuH7UOXscJw	8/19/16	181	70	37	-4	28	2:55	0	56
Little Boxes	Walk Off the Earth	iPhone	spotify:track:5jZkMJRsEu4naluH7VjiVB	7/6/15	120	40	84	-11	71	1:53	85	40
Little Fluffy Clouds	The Orb	IPod	spotify:track:7FVvHGA46aU7mkwx4iHMRE	4/2/91	105	54	66	-18	54	4:27	1	42
Livin' On The Edge	Aerosmith	Phone	spotify:track:4avSaixVwu8uqfSs7zgoj3	1/1/93	85	88	40	-4	27	6:21	0	57
Living Fantasy	Clark	iphone 6	spotify:track:21axGBCC9So7Hlfbfrvz3T	4/7/17	160	77	59	-8	67	4:07	12	28
Living In The Moment	Jason Mraz	ITunes	spotify:track:395XDt5vt8DKguJDjGtYLU	4/13/12	84	63	65	-7	70	3:55	5	53
Løb Stop Stå (feat. Coco O.)	Boom Clap Bachelors	Spotify	spotify:track:4SsNEB3KYHRyGrpJhi2Ddd	4/26/11	94	34	49	-14	28	4:49	86	45
Locked Out Of Heaven	Bruno Mars	Spotify	spotify:track:3w3y8KPTfNeOKPiqUTakBh	12/7/12	144	70	73	-4	87	3:53	5	79
Longe da te cor moi	Claudio Monteverdi	Spotify	spotify:track:5vgnbmKkimsNcQKCxfjoJf	1/1/99	67	2	17	-29	4	2:46	98	1
Look Wot You Dun	Steve Overland	Samsung tablet	spotify:track:50hSOIgrtK4	1/1/01	90	70	66	-8	95	3:14	18	8

			MlpURmgE89z									
Lord of Eternity	Fernando Ortega	I tunes on I mac	spotify:track:1HFTMNSMsDchQ50oTXhujK	8/5/14	161	41	39	-9	15	4:33	70	17
Lost	KT Tunstall	Spotify	spotify:track:4lQzPwJQKLryQjdHBFzE11	1/1/10	140	53	47	-7	18	4:41	33	24
Lost Mind (big band version)	Herman Brood	Laptop										
Love Gangster	Beth Hart	Spotify	spotify:track:5WF9Nvo2y3SpxlWvvibnmo	10/14/16	113	65	65	-5	37	4:10	9	33
Love Is a Stranger (Eurythmics)	The Plastic FanTastics	iPad	spotify:track:4qT2Tfk4TJir55vdh0YAZ4	7/4/11	127	79	63	-7	16	3:54	0	1
Love Is Stronger Than Justice (The Munificent Seven)	Sting	iPhone (iTunes)	spotify:track:2DXYjzsMEV6Kvb3Q61Y0M0	3/9/93	166	78	54	-8	78	5:11	6	34
Love Me Now	John Legend	Music on my iPhone	spotify:track:6nxQdXa1uAL0rY72wPZu89	12/2/16	124	76	49	-4	71	3:30	59	69
Love My Life	Robbie Williams	Laptop	spotify:track:1YW369EbVyjpeLE3YbsjKQ	11/4/16	96	69	54	-7	38	3:28	1	62
Love on the Weekend	John Mayer	Spotify	spotify:track:0j2WBxWZnWti5TpSxjJvPb	11/17/16	120	55	73	-9	41	3:33	55	67

Love The Fear - Tom Fall Remix	Temple One	Spotify	spotify:track:3Hp2oV0SDukUTcJunXC4QL	2/2/12	128	84	58	-4	36	7:00	1	4
Love The Way You Lie (Made Famous by Eminem and Rihanna)	Smooth Jazz All Stars	iPhone, music	spotify:track:6awsla6j83x2jFPpet4d1V	2/8/11	174	46	55	-10	33	3:40	67	16
Lover Boy	MIKA	Spotify	spotify:track:5u3i2xrCK6qC78dv1yX9zF	1/1/09	127	75	68	-7	72	3:13	45	27
Lovesick	Flume	Laptop										
Made In The USA	Demi Lovato	iTunes (on laptop)	spotify:track:4A4jWRGo7pRJ8hPLC9xVoG	1/1/13	88	87	58	-4	63	3:16	0	42
Making Christmas	The Citizens of Halloween	Spotify	spotify:track:208rmXdjs6mywkQRQw0xni	1/1/93	140	32	56	-16	28	3:58	68	29
Mama's Gun	Glass Animals	Deezer	spotify:track:5o4znEHwozOHIgHoWAllnr	8/26/16	90	31	46	-10	22	4:27	70	56
March Of The Witch Hunters - From "Wicked" Original Broadway Cast Recording/2003	Christopher Fitzgerald	Android/Google Play Music	spotify:track:1i74LECmLTK8uH3k2Z9BJa	1/1/04	115	67	58	-8	69	1:31	68	38
Mardy Bum	Arctic Monkeys	Spotify	spotify:track:2fyIS6GXMgUcSv4oejx63f	1/29/06	112	60	63	-5	31	2:55	3	72
Marilyn Monroe	Pharrell Williams	iPhone 6/Spotify	spotify:track:1AvXB7pTYb7irnDLRfE	3/3/14	117	59	88	-9	62	5:52	6	57

			27H									
Marvin Gaye (feat. Meghan Trainor)	Charlie Puth	iTunes	spotify:track:3MWGlCKyH4nILtXRnE9gd7	11/5/15	110	62	80	-5	82	3:10	42	54
Mary, Mary	Run–D.M.C.	Ipod Classic	spotify:track:3jmbbFdiHvAf1EGmveAHkU	9/16/88	115	88	89	-6	93	3:15	0	38
Me and Bobby McGee	Janis Joplin	Amazon Music	spotify:track:1IqFh00G2kvvMm8pRMpehA	1/11/71	93	46	45	-11	66	4:31	30	70
Memories (feat. Kid Cudi)	David Guetta	Spotify	spotify:track:5xYC48nOppVemY6U5GRGTb	11/22/10	130	92	55	-4	38	3:31	0	65
Mercy	Shawn Mendes	Spotify	spotify:track:0AS63m1wHv9n4VVRizK6Hc	4/20/17	148	66	56	-5	36	3:29	13	80
Mesmerise	Temples	Spotify	spotify:track:7H2MzfDoJVIhDUuWfDsaRQ	2/10/14	136	78	38	-6	52	3:42	0	34
Miracle of Life	Walk on Fire	AGPTEK MP3 player										
Miss Right	Anderson .Paak	Spotify	spotify:track:4it9okpXUNL0haiYRxfU0q	8/28/15	96	53	82	-7	71	3:19	0	51
Missing You	All Time Low	Spotify	spotify:track:0c2yZp1tTYAUiKraMdS8Yg	4/3/15	100	73	56	-6	40	4:05	5	64

Mixed Drinks About Feelings	Eric Church	Samsung S6 Music	spotify:track:1EnoCmQSpJaiCyA3Kr87XG	11/11/15	136	22	71	-13	42	2:58	52	54
Mo Hotta Mo Betta	The Baseballs	Ipod	spotify:track:3M0kMeCRbeo3SHbr9CKc2t	3/7/14	82	83	64	-5	96	3:01	47	11
Money Power Fame - Remastered	DON BROCO	Ipod Classic	spotify:track:1Ue0sFVGnKM8N8PUeFYMFW	8/7/15	97	89	61	-3	69	3:23	0	45
Monkey Wrench	Foo Fighters	Windows Phone/Groove Music	spotify:track:44wXefe8WB9Fd6xwtmAwbR	5/20/97	174	95	40	-4	56	3:51	0	68
Monsters of Sunderland	British Sea Power	Spotify	spotify:track:1E4JCH45a0Hk5wZeVUh2wW	4/1/13	173	74	30	-6	38	3:10	1	15
Moonbeam Levels	Prince	I pod	spotify:track:4i209ifm3SMgm9AQy8aMTY	11/22/16	76	51	61	-10	51	4:06	63	28
Morning	Beck	Spotify	spotify:track:65tH2GPMFCt8JtM2PM3MiX	1/1/14	114	36	55	-10	27	5:20	47	54
Motivational Speaker	Cowtown	Spotify	spotify:track:3t7eqck05Ir4QoSDbTmTzu	8/19/16	98	89	49	-8	76	2:35	0	7
Mountain To Move	Nick Mulvey	Spotify	spotify:track:7zGyIkunvMO11FByE93lzZ	9/8/17	110	95	66	-6	31	3:56	43	56

Move Down South	James	Apple ipod touch 6th generation	spotify:track:2TGCmCvCNy3pnWA9WxwqCu	3/18/16	135	84	45	-6	28	5:19	0	28
Moves	Big Sean	Spotify	spotify:track:0Fv5N0cHBsl4bzCbollCAS	2/3/17	76	53	79	-6	37	2:23	5	73
Movies	Alien Ant Farm	Spotify	spotify:track:0DVxWV6V1nWa7Ml68Frd3D	1/1/01	113	92	55	-5	53	3:16	0	59
Mr. Brightside	The Killers	Ipod	spotify:track:3n3Ppam7vgaVa1iaRUc9Lp	1/1/04	148	92	36	-4	24	3:42	0	81
Mr. Brightside	The Killers	Phone	spotify:track:3n3Ppam7vgaVa1iaRUc9Lp	1/1/04	148	92	36	-4	24	3:42	0	81
Mr. Brightside	The Killers	Spotify	spotify:track:3n3Ppam7vgaVa1iaRUc9Lp	1/1/04	148	92	36	-4	24	3:42	0	81
Mr. Brightside - Jacques Lu Cont's Thin White Duke Mix	The Killers	ITunes	spotify:track:46gog9DTymoam1Atq4xpDl	1/1/07	134	74	56	-7	39	8:48	1	47
Mrs. de Winter bin ich	Wietske van Tongeren, Susan Rigvava-Dumas	Ipod	spotify:track:2iK1Wm97NFurf0rfe9rsNe	11/17/06	77	49	53	-6	43	2:30	83	12
Mrs. Robinson	Simon & Garfunkel	Samsung S7	spotify:track:0iOZM63lendWRTTeKhZBSC	4/3/68	92	46	61	-14	81	4:04	71	74

Multilateral Nuclear Disarmament	Beastie Boys	Spotify on Desktop	spotify:track:18IFn8zywIcYcXmNP6ySI4	5/3/11	91	75	81	-6	72	2:54	1	36
Mundy: Vox Patris caelestis - 1. Vox Patris caelestis	William Mundy	iTunes	spotify:track:0fb8tDga0cmToQ0rLzs4ej	1/1/80	95	5	20	-31	4	2:16	99	9
My Boo	Usher	Apple	spotify:track:68vgtRHr7iZHpzGpon6Jlo	3/19/04	86	51	66	-8	68	3:43	26	77
My Lighthouse	Rend Collective	Spotify	spotify:track:0DemtmNC6rd3xt1zwkH84o	3/17/14	110	81	55	-6	73	3:44	3	44
My Michelle	Guns N' Roses	I pod	spotify:track:2dyfo7lqKI7NtSAhUZwnoJ	1/1/87	103	94	44	-8	31	3:39	17	57
My Name is Victory	Jonathan Nelson	Apple Music	spotify:track:5SDnSsuRRQXmIIU75neTAG	2/1/08	174	88	46	-5	65	6:32	23	44
My Pledge of Allegiance, Pt. 1: The Sealed Fate	After Forever	YouTube	spotify:track:6NObhCM6j1KpVffUlKP8L0	12/19/13	86	78	37	-7	12	6:24	17	27
My Silver Lining	First Aid Kit	Laptop/Spotify	spotify:track:5BkNCuxzzid0gz9sx3NNbX	6/6/14	102	73	48	-5	43	3:35	3	63
My Sweet Lord	Hurray For The Riff Raff	Spotify	spotify:track:0nCg55PB9EjzlXrc7Pms0Q	7/2/13	111	40	36	-10	58	4:01	79	38
My Young Man	Kate Rusby	iPod										

Naked as We Came	Iron & Wine	Deezer	spotify:track:7be9dRbxAmRogtGDJCVhzE	3/23/04	172	21	37	-17	66	2:33	63	49
Nandemonaiya - Movie Version	RADWIMPS	iphone	spotify:track:5RuenvbahShQoSVlH4mlKT	9/29/16	168	46	49	-6	51	5:42	24	35
Nasty	Kid Ink	Spotify	spotify:track:7CGl5DRfDKgVpoHZv1Thvj	6/3/16	126	60	69	-7	58	3:39	63	53
Native Sons	Snarky Puppy	Phone / Spotify Premium	spotify:track:6mhbgeO7a6AFFlvMCUaDEw	1/1/07	124	55	54	-13	26	8:54	7	26
Nausicaa Suite	Joe Hisaishi	iTunes										
Nautical Disaster	Rockabye Baby!	iPod	spotify:track:2F8oVH3ZZAiWhZNaqXiTP4	11/8/11	116	14	79	-17	58	3:23	92	18
Need The Sun To Break	James Bay	Spotify	spotify:track:5qrG9Ea6hen3VavTg9vQz9	3/23/15	152	49	54	-8	29	3:45	10	57
Neighbors	J. Cole	Spotify/Laptop	spotify:track:0utlOiJy2weVl9WTkcEWHy	12/9/16	139	28	89	-13	41	3:37	36	75
Neon Brother	Nothing But Thieves	Spotify	spotify:track:3onvqHzdPZXZrPo5GfLqGE	10/16/15	77	60	43	-5	15	3:56	1	50
Never Be Like You - Remix Flume & Kai Tribute	Farbwall	Spotify/Phone	spotify:track:3MVf1n674dAGnXEGaG	6/20/16	128	63	82	-7	56	3:18	20	12

			uTK6									
Never Ever - Radio Edit	All Saints	Spotify web player - desktop PC	spotify:track:4JmS637roUkZqCjUjn030K	3/25/16	134	47	73	-6	33	3:54	75	55
Never Forget You	MNEK	Spotify	spotify:track:6v4F5IaKUITBPzt2wztoiZ	7/22/15	146	73	58	-6	28	3:33	0	55
Never Hurt Again	Aquilo	Spotify	spotify:track:1XxUhyChC3lauYQ4BINva2	1/27/17	110	60	85	-8	49	3:00	5	56
New American Times	James Moye	Android/Play Music (Google)	spotify:track:7I3Pxz8dWv7GvYNIgafIY4	11/26/13	95	42	52	-5	32	3:26	85	2
Next Girl	The Black Keys	ipod	spotify:track:2NdNUzldjsnx6cq6Qo2vlo	5/18/10	86	70	52	-6	24	3:18	4	47
Next Year - Live	Foo Fighters	Spotify	spotify:track:1TQ9HF8LMlOTAIJeX472hk	11/7/06	174	87	19	-4	61	4:35	0	41
Nightfall	Xandria	Spotify	spotify:track:2uyHShRKvdoT9yBCS5Yzrl	5/5/14	172	88	40	-6	17	3:55	1	22
Nights With You - Cheat Codes Remix	MØ	Iphone	spotify:track:2lE6CIzmQvcF7Mo3z4k9cr	6/9/17	116	80	74	-6	46	4:06	0	60
NO EXCUSES	Meghan Trainor	Spotify	spotify:track:7fCNUWi6uflDTQ08srxM	3/1/18	115	66	83	-4	52	2:33	2	79

			Zk									
No Problem (feat. Lil Wayne & 2 Chainz)	Chance the Rapper	Spotify	spotify:track:0v9Wz8o0BT8DU38R4ddjeH	5/27/16	135	80	65	-5	79	5:05	16	78
No Problem (feat. Lil Wayne & 2 Chainz)	Chance the Rapper	Spotify	spotify:track:0v9Wz8o0BT8DU38R4ddjeH	5/27/16	135	80	65	-5	79	5:05	16	78
Not With Haste	Mumford & Sons	iPhone	spotify:track:2rC0gZUp3Flzus9OtZtnDP	9/21/12	78	48	32	-9	12	4:07	67	38
Nothing's Wrong	HAIM	Deezer	spotify:track:0rurOLe6Zi1jl1fbf586vW	7/7/17	144	73	55	-5	62	3:09	6	51
Now and Later	Sage The Gemini	Spotify	spotify:track:43jBqV3j3Xi1g6wO0bhIMd	10/14/16	107	57	78	-6	86	3:13	27	67
Now I'm Freaking Out	Ween	iTunes										
Now Or Never	Stuart Valentine	iPod Nano	spotify:track:5GMQ7IV8XclbMgvl6O9eQv	10/2/07	100	70	51	-9	52	2:47	41	0
Objection (Tango)	Shakira	Sansa Clip Sport	spotify:track:36YNa8joLwu9yor2TkZbIY	11/12/01	179	86	60	-5	71	3:43	1	53
Of Kali Ma Calibre	Diablo Swing Orchestra	Computer	spotify:track:718bClZmBl2Nul32U20mfJ	5/14/12	180	91	25	-4	60	4:25	1	24

One day more	Les miserable 10th Anniversary	I phone. Itunes										
One More Night	Maroon 5	iPhone Music app	spotify:track:6cpk00i5TxCqSeqNi2HuIe	1/1/12	93	82	72	-3	62	3:40	6	70
One More Night	Maroon 5	Iphone	spotify:track:6cpk00i5TxCqSeqNi2HuIe	1/1/12	93	82	72	-3	62	3:40	6	70
One Take Freestyle	Stormzy	Spotify	spotify:track:4Aqk447OISHIGHP7YJaSF4	9/2/16	88	82	65	-6	92	2:50	22	51
One Thing Remains	Brian Johnson	Spotify	spotify:track:0nQGfkhI2n3KlcJomfWnyx	3/22/11	148	85	38	-7	28	5:01	0	45
Only my railgun	marasy	iTunes										
Only Our Rivers Run Free	Riogh	iphone										
Only The Lonely - Remastered	Chris Isaak	iPhone / music	spotify:track:1NLQRBxYvRthEbj3v0cXAG	5/5/06	113	25	58	-11	45	2:53	89	27
Other Woman	Paloma Faith	IPhone 6 / Music	spotify:track:0FbuGQu6L5jSxGGxHo98Zx	11/7/14	131	78	73	-4	89	3:09	34	38
Overture	Andrew Lloyd Webber	Amazon Music App	spotify:track:7o3DV1szMH5Fd38f1QwgPO	12/10/04	121	43	18	-12	28	2:47	0	48
Paint It Black	The Rolling Stones	iPod	spotify:track:0wzABO1igQsSy8cQ7dI	4/15/66	159	80	48	-9	57	3:22	6	72

			eHK									
Paint You Wings	All Time Low	Spotify	spotify:track:6NMzAIKmif0HbMREXBuiZ4	9/30/13	160	98	42	-4	57	3:40	0	45
Painting the Clouds with Sunshine	Jack Hylton & His Orchestra	iTunes	spotify:track:3e5d8kQU22qtHgkHbr5Orm	10/1/13	95	23	80	-18	89	2:59	98	17
Panic Tree	Jimi Goodwin	Spotify	spotify:track:5M57vu5a2B9a1WoaDijjhS	3/24/14	124	70	59	-6	79	2:37	1	4
Paradise	Vaults	Spotify	spotify:track:7haB8dccoBnqkJhQHBuiLo	12/2/16	81	55	30	-7	14	3:07	2	30
Paradise City	Guns N' Roses	iPhone	spotify:track:3YBZIN3rekqsKxbJc9FZko	1/1/87	100	95	27	-9	48	6:46	2	77
Paranda	Kaur-B	Phone	spotify:track:5QCE1mbxB3ugEM7IMWK9Yq	7/10/18	90	83	79	-3	77	4:07	38	39
Paris	The Chainsmokers	Deezer	spotify:track:15vzANxN8G9wWfwAJLLMCg	1/13/17	100	64	65	-7	25	3:42	2	78
Paris	The Chainsmokers	Spotify	spotify:track:15vzANxN8G9wWfwAJLLMCg	1/13/17	100	64	65	-7	25	3:42	2	78
Part 11	Philip Glass	Spotify	spotify:track:6Xmig32xALjYPqPaDD	4/23/13	121	34	18	-15	19	14:30	95	6

			WtLi									
Party Like a Russian	Robbie Williams	iPod touch	spotify:track:5jvAliGepxzKOhGp01o5IT	11/4/16	148	91	49	-5	36	3:03	1	53
Peace Piece	Bill Evans	iTunes	spotify:track:58yFroDNbzHpYzvicaC0de	1/1/59	133	3	46	-20	7	6:44	99	61
Pelican	The Maccabees	Apple Music on iPhone	spotify:track:0HjRzOti4wvuKwIUsjZrHM	1/1/12	184	91	39	-4	58	3:45	19	44
Penny Lane - Stereo Mix 2017	The Beatles	Spotify	spotify:track:57YeN4L1zAYvnIlLwL3hp6	6/1/67	113	54	62	-8	64	2:59	4	65
People Ain't No Good - 2011 Remastered Version	Nick Cave & The Bad Seeds	iPhone	spotify:track:0c9ImAFCxarmltq6a1jCSe	1/1/97	120	11	43	-17	23	5:42	86	52
Personal Jesus - Single Version	Depeche Mode	Spotify	spotify:track:4UCTgh5jvtqoaMX6MAhUNn	9/28/98	130	90	67	-4	79	3:45	9	67
Piano Man	Billy Joel	Spotify	spotify:track:70C4NyhjD5OZUMzvWZ3njJ	11/7/73	178	55	33	-6	43	5:39	61	67
Pièces de clavecin en concerts - Concert No. 3 - iii. Tambourins	Gustav Leonhardt	Spotify	spotify:track:6ql69cgeGj8KXxGWiz9bIg	6/12/12	130	46	50	-12	88	2:54	54	0
Pitch Black	Vicetone	Mobile phone / Google play	spotify:track:1tTNmSHxNX1ZrM44apI	1/22/16	128	87	62	-7	38	2:55	1	44

		music	g53									
Play That Funky Music	Wild Cherry	Spotify	spotify:track:5uuJruktM9fMdN9Va0DUMl	1/1/76	109	67	81	-12	93	5:00	4	67
PLAYING WITH FIRE	BLACKPINK	Spotify	spotify:track:7e7VjLxO5xJINHvnRytrqi	11/1/16	97	76	71	-5	67	3:17	4	75
Pocket Calculator - 2009 Remastered Version	Kraftwerk	iTunes	spotify:track:4xa1gzSKd6ihtVGd8dWs69	1/1/81	136	46	86	-13	96	4:57	63	42
Poison	Alice Cooper	MP3	spotify:track:5XcZRgJv3zMhTqCyESjQrF	7/25/89	119	91	28	-6	26	4:30	3	68
Polar Bear	King Charles	Phone (Vodafone Smart Ultra 6) on play music app	spotify:track:6LrYjqcs6TaEZoRUKnfULu	1/1/12	170	76	51	-7	54	4:32	8	17
Porcelina of the Vast Oceans	The Smashing Pumpkins	Apple music	spotify:track:2I0r4awu38uuWfpEuH35mH	12/12/95	111	44	31	-11	5	9:22	0	45
Prelude	I Am Abomination	Itunes	spotify:track:1NvcDo7Am6GXbLO9sUlOee	1/1/09	160	91	34	-6	46	0:48	16	9
Princess of China	Coldplay	iPhone	spotify:track:4HXOBjwv2RnLpGG4xWOO6N	10/24/11	85	69	43	-6	23	3:59	0	68

Promenade	Keston Cobblers Club	Iphone	spotify:track:5TekJZoBG4rRYl9LtIW52u	8/27/12	125	27	68	-12	29	1:21	99	10
Promise	Ben Howard	Spotify	spotify:track:4qyfir5Yr7nfo05g6cyFMT	1/1/11	157	19	37	-16	18	6:24	89	63
Psycho	Muse	iPhone 7 / Apple Music	spotify:track:383QXk8nb2YrARMUwDdjQS	6/4/15	125	88	56	-3	51	5:17	0	70
Psychotic Reaction	Count Five	Spotify	spotify:track:5JcaA4A9ZoXthwEnxOxWvP	1/1/66	165	75	35	-7	67	3:06	0	53
Pulcinella Suite: VII. Vivo	Igor Stravinsky	Spotify	spotify:track:3B0uJXOUzCVrSQmAJmxPA1	6/3/16	127	18	66	-18	76	1:31	98	13
Pumped Up Kicks	Foster The People	Spotify	spotify:track:7w87IxuO7BDcJ3YUqCyMTT	5/23/11	128	71	73	-6	97	4:00	14	83
Purcell / Arr Pluhar: Oedipus, King of Thebes, Z. 583/2: "Music for a while"	Henry Purcell	Spotify	spotify:track:2UK3kMSQc8fMTCjEykifQ7	2/26/14	137	5	36	-23	12	5:54	96	36
Pure Morning	Placebo	iPhone 6s	spotify:track:1TqmZVIyeD4aN4Wa0wbnfy	10/12/98	81	90	39	-7	20	4:15	4	60
Purple Rain	Prince	iTunes	spotify:track:54X78diSLoUDI3joC2bjMz	6/25/84	113	45	37	-10	19	8:41	4	75
Put the Blame On Mame - Theme from	Rita Hayworth	Apple Music /	spotify:track:1G6kNAmm	2/14/14	111	26	53	-16	79	2:18	99	28

"Gilda" Original Soundtrack		Iphone	AE4c75OiN UXVqT									
Questing, Not Coasting	Maximo Park	Ipod classic	spotify:track: 5XDkyRWa GN3mrpw11 lfVGa	10/5/09	150	83	43	-6	49	3:42	9	29
Question Is	Tory Lanez	Apple Music	spotify:track: 5bhAv8JVL W77s3VjxGt 4Q6	8/19/16	80	58	64	-8	46	5:05	64	44
Quit Playing Games (With My Heart)	Backstreet Boys	Spotify web player - desktop PC	spotify:track: 0Uqs7ilt5kG X9NzFDWT BrP	1/1/96	100	83	80	-7	90	3:53	6	65
Radio Silence	James Blake	Iphone	spotify:track: 0ZPPbwJD2 WVU8HdMs VtChk	5/5/16	120	41	53	-10	42	4:01	77	50
Radioactive	Imagine Dragons	Spotify	spotify:track: 62yJjFtgkhU rXktIoSjgP2	1/1/12	136	79	46	-4	20	3:07	11	78
Rainingmen	Sister sledge	iPhone										
Rather Be (feat. Jess Glynne)	Clean Bandit	iTunes	spotify:track: 0TVV2gFRO JaB3kIZyCU vIY	5/27/14	121	59	80	-7	55	3:48	16	61
Read all about it	Emeli Sande and Eminem	Apple music										
Reasons Not to Be an Idiot	Frank Turner	Ipod	spotify:track: 20InRAJs767 clBlHMhpn Wz	3/30/08	145	63	55	-11	79	3:49	0	32
Red Light Indicates Doors Are Secured	Arctic Monkeys	iTunes	spotify:track: 56GFlp1dkt7 dw56pQdTw	1/29/06	111	85	62	-5	91	2:24	0	58

			qW									
Rehab - Live On Jools Holland	Amy Winehouse	Spotify	spotify:track:7E4dtl5gWROiMKibvedh0i	10/30/15	144	78	43	-5	43	3:41	4	49
Rejoice (Live)	Sonnie Badu	Spotify	spotify:track:3Ym7gwmNWzHdJBs9hNMEtJ	9/21/16	89	86	57	-4	63	5:05	47	21
Remember The Name (feat. Styles Of Beyond)	Fort Minor	Spotify.	spotify:track:6ndmKwWqMozN2tcZqzCX4K	1/1/05	85	84	69	-4	88	3:50	6	78
Reminder	The Weeknd	iPhone	spotify:track:37F0uwRSrdzkBiuj0D5UHI	11/25/16	160	51	71	-7	39	3:39	14	74
Revelation Song - Live	Passion	Spotify	spotify:track:35msHwn81uKQS5Jn783oSU	1/1/13	127	56	21	-6	9	8:29	4	52
Revolting Children	Matilda the Musical Original Cast	Ipod	spotify:track:5jb55O3h5dfOK4YMUbTb1Y	10/13/11	116	70	72	-6	59	2:33	47	56
Revolting Children	Original Broadway Cast Of Matilda The Musical	Ipad	spotify:track:4xwyLdkNblvpSqGRej9at0	9/26/13	116	70	68	-8	57	2:33	47	29
Riptide	Vance Joy	Ipod	spotify:track:7yq4Qj7cqayVTp3FF9CWbm	9/8/14	102	73	48	-7	51	3:24	43	77
River	Joni Mitchell	ITunes	spotify:track:0DAmSYQW9kq9gQN	6/22/71	61	15	41	-14	38	4:05	98	54

			DI002KP									
Rock Bottom	Neck Deep	Spotify	spotify:track:1v6tmbdVrsUylFyP2b81Jg	8/14/15	105	93	58	-4	53	3:21	0	51
Rockabye (feat. Sean Paul & Anne-Marie)	Clean Bandit	Laptop	spotify:track:5knuzwU65gJK7IF5yJsuaW	10/21/16	102	76	72	-4	74	4:11	41	84
Rocker - Remastered	Miles Davis	Spotify	spotify:track:7seafSAoX3q3WSnllk0nvI	1/1/57	102	33	48	-12	43	3:07	73	31
Rolling in the Deep	Adele	iphone	spotify:track:1eq1wUnLVLg4pdEfx9kajC	1/19/11	105	77	73	-5	51	3:48	14	73
Romeo and Juliet	Steve Knightley	Ipad	spotify:track:3AC0kCOoj6RctDbgPuyDZ9	6/4/07	79	47	45	-12	31	6:11	34	12
Room to Breathe	You Me At Six	iPod	spotify:track:5jhbFrDtzcICeCfYzxUvHo	1/27/14	143	93	42	-3	29	3:59	0	43
Rootless	Marina and the Diamonds	iPhone 5S (Music app)	spotify:track:3ZbMNfEbxMPNxNPOFPxrqj	2/15/10	114	81	61	-7	68	3:29	6	39
Rose Of Sharyn	Killswitch Engage	Spotify	spotify:track:3ZlleUP5EE2rixueLVCi2M	5/3/04	91	98	47	-4	41	3:36	0	45
Roulade	Ken Cowan	ITunes										

Royals	Lorde	Mac	spotify:track:2dLLR6qlu5UJ5gk0dKz0h3	1/1/13	85	43	62	-10	30	3:10	11	81
Royksopp's Night Out	Röyksopp	Spotify	spotify:track:2cxyrWGI8Yy5JowrTvFwTR	1/1/01	101	88	67	-8	76	7:30	1	23
Ruidos	Cuarteto Cedrón	iPhone/Music	spotify:track:6njiQX9G49PrWWTfqtcW6J	10/23/12	82	35	29	-6	33	3:32	61	1
Rumor Has It	Judson Mancebo	Phone	spotify:track:0tX9fzRmoOiTo1orsStTsp	6/27/18	96	18	64	-17	33	3:51	99	10
Running On Faith	Eric Clapton	Spotify Laptop	spotify:track:64fZ1gcn7rEzbdibOP1inl	8/25/92	131	56	54	-9	52	6:31	22	50
Ryo	Kawabata Makoto	iPod Classic	spotify:track:142pBoyrBvJkaSvrFyI4RM	9/18/07	134	66	15	-10	3	67:51:00	4	0
Sally free and easy	Show of Hands	ipod										
Santa Monica Dream	Angus & Julia Stone	Spotify	spotify:track:47YITQYhJXw79nCor48tnq	3/15/10	120	26	47	-14	7	5:30	91	53
Save Me	BTS	Spotify	spotify:track:54s6sdPuMjVtdB5d5KFzTC	5/2/16	140	82	61	-3	54	3:17	3	75
Savior	Rise Against	Spotify	spotify:track:1vcxF91pWs9uNwDROuiCPB	1/1/08	112	93	55	-3	48	4:02	0	73

Say You Won't Let Go	James Arthur	Spotify	spotify:track:1Pw5C4N6Fn5E4mGCxmbbVa	9/9/16	99	56	40	-7	48	3:31	69	80
Sea of Love	Cat Power	Spotify	spotify:track:1J3w85cS3FEmoSKRu2dQJ8	3/21/00	78	9	56	-11	12	2:19	94	62
Seasons Of Love - From The Motion Picture RENT	Cast Of Rent	I phone / I tunes	spotify:track:3SnXIPWciHwAS04oKdPC0l	8/2/05	178	57	44	-5	44	3:04	58	53
Seasons Of Love - From The Motion Picture RENT	Cast Of Rent	Samsung S6	spotify:track:3SnXIPWciHwAS04oKdPC0l	8/2/05	178	57	44	-5	44	3:04	58	53
Seb-1.02	Squarepusher	Spotify	spotify:track:5r58grBRpMoQEb0bieoXLG	1/1/09	103	6	45	-21	20	1:58	96	9
Second Hand News - Remastered	Fleetwood Mac	iPod	spotify:track:0hlniElQOr5tvSKBaSpAQi	2/4/77	118	94	70	-5	50	2:56	24	58
Secret Heart	Feist	iPod	spotify:track:7JVd7kn6A62mGzlr03iNTE	1/1/04	101	43	78	-15	36	3:49	85	31
Secret Someones	Laura Veirs	Spotify	spotify:track:1poF1ikXHBFF2bGkvmYrmI	8/23/05	143	71	49	-9	46	5:15	9	19
Seein' Stars	Speaker Of The House	Spotify	spotify:track:1IoiRzGCfRGc8fAPgqn7z0	2/19/15	124	72	69	-5	32	4:47	17	27

Segull	Hugar	Spotify	spotify:track:5jb0HjJA7RvwrU5sFq0FM1	7/28/14	70	1	7	-30	4	3:29	98	11
September Song	JP Cooper	Spotify	spotify:track:0zbzrhfVS9S2TszW3wLQZ7	10/6/17	96	62	61	-7	37	3:40	5	74
Seventeen Landscapes	Heron Oblivion	Spotify	spotify:track:5c6n2GFHftnwkFhAEQR08K	3/4/16	80	30	37	-11	19	7:14	58	2
Sex With The Ex	Reverend And The Makers	Spotify	spotify:track:32l6wURmcAQEXhIFMNFHLa	9/16/07	117	46	42	-6	7	4:07	1	29
Sexy Den A Mutha	Cheryl	Apple music	spotify:track:4erAL4EBoD3Yx6Dwx83oAB	1/1/12	129	86	74	-4	65	3:40	9	33
Shadows In The Moonlight	Anne Murray	iPad	spotify:track:5qujByYxjT21vCeRIaOoqI	1/1/94	105	56	66	-9	40	3:30	2	33
Shake Your Body (Down to the Ground) - Single Version	The Jacksons	Phone (Samsung Galaxy S6)	spotify:track:0bSTBRdN4iBepZ8bUcVq0S	6/28/04	117	73	80	-7	84	3:45	5	49
Shape of You	Ed Sheeran	Spotify	spotify:track:7qiZfU4dY1lWllzX7mPBI3	3/3/17	96	65	83	-3	93	3:54	58	90
Shape of You	Ed Sheeran	Spotify	spotify:track:7qiZfU4dY1lWllzX7mPBI3	3/3/17	96	65	83	-3	93	3:54	58	90

Shoes and Ships	Fuchsia	Itunes										
Shostakovich : String Quartet No.8 in C minor Op.110 : III Allegretto	Dmitri Shostakovich	Desktop computer/Spotify	spotify:track:0ESJA9rZJGzdkRuGBrssdv	1/1/90	124	5	47	-25	49	4:05	96	8
Should I Stay or Should I Go - Remastered	The Clash	Spotify	spotify:track:39shmbIHICJ2Wxnk1fPSdz	1/1/82	113	83	74	-6	82	3:09	8	78
Shuffle	Bombay Bicycle Club	Laptop - Spotify	spotify:track:4yYvWFUWMaKMXtoJKJnmZk	1/1/11	110	96	63	-5	40	3:55	32	58
Shut Up and Dance	WALK THE MOON	iPad	spotify:track:0kzw2tRyuL9rzipi5ntlIy	6/15/15	128	87	58	-4	62	3:19	1	68
Sibelius Violin Concerto	Victoria Mullova	iphone										
Side to Side (Originally by Ariana Grande and Nicki Minaj) - Karaoke Instrumental	Karaoke Freaks	Spotify	spotify:track:6A9lUa9ugPmAOap2sDQ89d	9/1/16	161	28	81	-14	32	3:37	4	4
Simple Song	Passenger	I pad	spotify:track:70HWRkitkAKfos500LBk0T	7/28/17	78	40	63	-11	60	3:48	62	65
Singet dem Herrn ein neues Lied, BWV 225: Lobet den Herrn in seinen Taten	Johann Sebastian Bach	iTunes	spotify:track:26HsPAGeQN6KLnXQhmezum	6/5/12	102	33	45	-22	72	3:09	99	16
Sink To The Bottom	Fountains Of Wayne	Samsung S6 Music	spotify:track:32uXfxJERzv4RK1gMLXbOA	1/1/96	97	71	47	-6	81	3:12	1	43

Sitting on the Dock of the Bay - Live	Percy Sledge	ipod	spotify:track:46rT4C0rDU2blIj6aNksql	3/10/17	174	57	37	-8	22	3:25	43	14
Six Billion	Nothing But Thieves	Spotify	spotify:track:44yES5bd4ezm4W6workRfh	10/16/15	144	45	30	-6	21	3:47	15	51
Six Feet Under	The Weeknd	Apple Music	spotify:track:4mU5iXHeLgbR94siF7p1sY	11/25/16	140	50	77	-8	24	3:58	13	70
Six Weeks	Of Monsters and Men	Phone, Amazon Music	spotify:track:29uPSxuWuR6Hr1SUu2Cw6z	1/1/12	124	88	38	-6	42	5:34	0	51
Sleepless Nights - GANZ Remix	Hamertje Tik	Spotify	spotify:track:62V56f4QO63mMuhGKv0fRO	5/26/14	137	70	64	-9	63	4:19	0	23
Slip Away	Mad Season	Apple music	spotify:track:34jo30lPEqp6QvJe04NAFu	1/1/95	116	57	31	-6	13	5:38	0	42
Slunecko	Cechomor	Spotify	spotify:track:2s2YWEQPbJAwOhZs57NnuW	1/1/00	160	75	52	-7	39	3:54	20	10
Smile	Otxhello	Soundcloud										
Smooth	Santana	Music (Iphone)	spotify:track:0n2SEXB2qoRQg171q7XqeW	1/1/99	116	92	61	-4	96	4:55	16	71
Smooth	Santana	Apple iphone	spotify:track:0n2SEXB2qoRQg171q7XqeW	1/1/99	116	92	61	-4	96	4:55	16	71

Smooth	Santana	Ipod classic	spotify:track:0n2SEXB2qoRQg171q7XqeW	1/1/99	116	92	61	-4	96	4:55	16	71
So What	P!nk	Samsung tablet	spotify:track:19qn6oU2t0E72ENA0aWNsX	1/1/08	126	89	54	-3	45	3:35	0	70
So You Want To Be A Rock 'N' Roll Star	Patti Smith	iTunes	spotify:track:5KGJghq19EmdyeLbtceyRm	1/1/01	149	80	25	-6	64	6:03	51	30
Social Disease - Remastered 2014	Elton John	iPod	spotify:track:0Gvc5Ii3duAifGy2QaFDVt	10/5/73	190	79	35	-7	82	3:43	22	34
Sofia	Alvaro Soler	Spotify	spotify:track:0W2pKKNHnfZZgcAxDPW0hy	7/15/16	128	73	70	-6	89	3:30	12	60
Someone In The Crowd - From "La La Land" Soundtrack	Emma Stone	Spotify	spotify:track:39ncDMVidHOeQgeC5anYZM	12/9/16	128	54	62	-9	28	4:20	9	65
Something Good	alt-J	Spotify	spotify:track:4JVSkSJAKbSlwkSepkXcK6	5/28/12	109	59	68	-10	52	3:38	73	57
Something That We're Not	Demi Lovato	Itunes	spotify:track:527vaaTSgrFZ23x8CGA48f	1/1/13	129	94	74	-3	88	3:17	3	33
Sommarfågel	Wintergatan	Iphone/Spotify	spotify:track:3fny5rAub4a4UAOiEo3Tny	4/24/13	180	87	36	-7	35	3:53	10	42

Song 2 - 2012 Remastered Version	Blur	Spotify	spotify:track:1FTSo4v6BOZH9QxKc3MbVM	2/10/97	130	79	67	-7	92	2:01	0	77
Song With a Mission	The Sounds	Iphone	spotify:track:5WC7VHPijgtfOlQrvmtaZr	3/15/06	150	75	54	-3	80	2:58	2	37
Soon After This	Broken Twin	Iphone	spotify:track:1peNmmft2qHn3KFgDQZatJ	4/29/14	84	8	37	-16	8	4:30	90	12
Soon and Very Soon	Selah (American Gospel Group)	Ipod Nano										
Sound & Color	Alabama Shakes	Mac	spotify:track:4jVQBszyxsa0GeRSe5ToVC	4/17/15	115	43	55	-7	18	3:02	85	57
South Wales and West : Television March (Original title Seven Seas March) (Recorded 1937)	Eric Coates	default music player on LG Android phone	spotify:track:0mkw1t4tP3pY7sJwGSAwdK	7/1/13	134	46	49	-16	67	2:48	55	0
Southern Cross	The Ozark Mountain Daredevils	iPad	spotify:track:69hbKkOa45vh2oWOwDeBFG	1/1/75	140	78	53	-11	78	3:30	2	11
Spread Your Wings - Remastered 2011	Queen	Zune	spotify:track:0nUCaKwNqO5whVAhEX1A1R	10/28/77	175	66	40	-6	49	4:34	29	54
St. Paul's	Deaf Havana	Spotify	spotify:track:0dMOQ7nZAx7JV7M6nFhRzM	1/27/17	108	52	38	-7	15	3:58	24	38

Stand Up	Dakoda Motor Co.	iPhone/Spotify	spotify:track:2bgy5rVeT3VrVCrtQg16z0	1/1/94	132	94	59	-5	73	4:21	2	6
Starboy	The Weeknd	Spotify	spotify:track:7MXVkk9YMctZqd1Srtv4MB	11/25/16	186	59	68	-7	49	3:50	14	84
Starry Eyed	Ellie Goulding	Spotify	spotify:track:14OF4JQOIm4hb0GfG5nfyF	1/1/10	150	81	50	-5	60	2:57	14	57
Stepson Of The Clapper	J Dilla	Ipod classic	spotify:track:4fy2Asi2h2BrzpzipU8zWl	2/7/06	99	68	62	-8	19	1:01	5	37
Stille Nacht, heilige Nacht	Franz Xaver Gruber	iPod	spotify:track:0dGoDyJ0PFAR4hPXc4niKK	1/1/16	77	16	17	-18	4	3:24	99	0
Storm	Ruelle	iPhone, music										
Streets of London	Ralph McTell	Iphone/Spotify	spotify:track:0P49pJa288tm8gKbFcofn9	1/1/69	156	30	40	-15	19	4:09	63	53
Stressed Out	Twenty One Pilots	Samsung Music	spotify:track:3CRDbSIZ4r5MsZ0YwxuEkn	5/15/15	170	64	73	-6	65	3:22	5	84
String Quartet No. 10, "Hosttidlos" (Harvest Timeless): Allegro fluente	Per Nørgård	Spotify	spotify:track:4Wr28Ded0IqqlHkltqRMrI	5/1/08	78	7	22	-24	4	13:40	96	18
String Quartet No. 7 in F Major, Op. 59/1, "Razumovsky Quartet No. 1": I. Allegro	Ludwig van Beethoven	Spotify	spotify:track:6Fs4kRAu4c39smFU43jvIc	4/29/14	149	12	32	-18	17	11:40	96	0

Subterranean	Foo Fighters	Ipod touch 6th generation	spotify:track:3O2de2JdIqnVVb5SBpg6wm	11/10/14	113	66	39	-7	6	6:08	0	48
Summer	Calvin Harris	iTunes	spotify:track:6YUTL4dYpB9xZO5qExPf05	10/31/14	128	86	60	-4	74	3:43	2	81
Summer Love	One Direction	Sony	spotify:track:2LjmbI7LJt0RDg612cBi7U	11/9/12	122	59	50	-5	29	3:28	36	60
Summer Music, Op. 31, First Movement, by Samuel Barber	St. Louis Symphony Orchestra	Zune										
Summer Nights	Florrie	iPhone 6 / music	spotify:track:01tEYiHPfq2A9pPm4z9lDZ	11/15/10	115	90	76	-4	88	3:55	1	23
Summer of 69	Danny Brian	Ipod	spotify:track:3hPpdYz6TPu4j9iVm9vrlr	1/20/15	139	59	52	-7	59	3:29	32	24
Summertime	Billie Holliday with Bob Haggart and His Orchestra	iPod / iTunes	spotify:track:4A8kiMOvOl8k4toAYzT4BO	3/20/17	99	20	62	-13	61	2:55	89	11
Summertime	Willie Nelson	IPhone	spotify:track:0zMw7VSIkgTxiP8dQSOHHi	2/26/16	100	27	55	-13	30	3:54	83	38
Sun Hits The Sky	Supergrass	Spotify	spotify:track:0mIZErjrNQzOTDtn4UYflo	4/21/97	151	93	37	-7	37	4:56	0	47
Sunshine Of Your Love - Live	Eric Clapton	IPhone	spotify:track:5JxU4Ke4oAQ4cyP4myFe	11/5/02	113	93	54	-4	48	7:11	7	33

			D3									
Sunshine On My Shoulder	Orquesta Música Maravillosa	Ipad	spotify:track: 0denJ7qYMpU2zBPeOSOoJW	5/11/15	148	34	60	-20	41	5:44	93	0
Super Rich Kids	Frank Ocean	Spotify	spotify:track: 0K9oqDmJBgSFjXU1bUY9Fk	1/1/12	60	42	74	-8	62	5:05	24	63
Superstitious	Stevie Wonder	I pod shuffle										
Surfin'	Kid Cudi	Spotify	spotify:track: 3f2k8op0nWDoZM4pXim6wG	9/30/16	130	69	81	-8	84	3:58	11	60
Suspended Animation	KMD	iPod Classic	spotify:track: 7reWvKIojKYlLEJVE64eqI	12/18/15	90	67	69	-9	50	2:24	8	22
Sweet Mood	Dennis Wilson	iTunes										
Sweet Talk	Dear and the Headlights	Spotify	spotify:track: 757fXABDTbalNzihYS3mUc	1/1/07	161	91	43	-6	74	2:57	0	48
Sweet Talk	The Killers	Samsung phone/Google play music app	spotify:track: 4IBTEftHDFMV8femNMlfMP	1/1/07	180	92	37	-4	39	4:19	1	51
Swings And Waterslides	Viola Beach	Spotify	spotify:track: 0RPlftkdYfx8YUJ0F671iM	7/29/16	120	79	64	-5	26	3:39	1	51
Symphony No. 3: II. Allegro molto	Aaron Copland	Spotify	spotify:track: 4HK6fU55Q	6/9/17	76	12	37	-17	20	8:36	96	23

			3kTyr4tyAM 58x									
Symphony No.39 In E Flat, K.543: 3. Menuetto (Allegretto)	Wolfgang Amadeus Mozart	Spotify	spotify:track: 5MvRYMK5 gWk65ppvZ wFgJ3	1/1/02	158	9	37	-19	37	4:23	99	41
T.I.N.A. - Originally Performed by Fuse ODG feat. Angel	Chart Nation	Itunes/Ipod	spotify:track: 4LgCPxyPE5 4MdOKcZTe HtE	10/19/14	127	96	66	-4	93	3:26	2	2
Tainted Love	Gloria Jones	iPhone - Spotify	spotify:track: 0BbA7S30m OQB94e4IB nqCt	1/1/82	161	69	52	-6	87	2:11	58	51
Tainted Love	Gloria Jones	Spotify	spotify:track: 0BbA7S30m OQB94e4IB nqCt	1/1/82	161	69	52	-6	87	2:11	58	51
Take Her To The Moon	Waterparks	Phone	spotify:track: 11y6CPvsQa 57dxfDQ0y3 CO	11/2/16	125	81	57	-4	71	3:15	1	34
Take Me There	Adore Delano	iTunes playlist	spotify:track: 1OicPC7Mx R2qPePqvN RPx9	3/11/16	125	84	60	-3	35	3:16	0	41
Take Our Time	TLC	iPhone/Mus ic	spotify:track: 44QrzYVAH FC9WMN91 GEo9H	11/15/94	142	56	65	-9	35	4:34	0	50
TALK ME DOWN	Troye Sivan	Windows music player on my phone	spotify:track: 1othhMi2Ws iF9oLoisJjY W	12/9/15	174	78	36	-7	35	3:57	15	61
Tavas domas	Jorspeis	iPhone	spotify:track: 0aVjQbQgm bRXUWHjV	6/18/12	160	65	27	-5	50	3:17	44	1

			BH48x									
Tears in the Rain	Triumph	ipod Nano	spotify:track:4opZJswPga1ZqItW19RAST	9/11/86	116	86	61	-3	76	3:55	4	28
Teenage Daughter	D.I.D	Spotify	spotify:track:1O8F1vfeTf7mxVtUhSx2GD	10/5/12	170	67	37	-6	16	4:13	0	31
Teenage Dirtbag	Wheatus	Amazon Music App	spotify:track:3LI4MmibTkXH5cGpCGZgyw	9/29/00	95	84	62	-4	57	4:02	36	71
Thank You For Saving Me	Delirious?	iTunes	spotify:track:32YJgZ5nZI6n3FgeHJRaU1	4/22/12	90	49	27	-10	39	7:02	27	20
That's Life	Michael Bublé	iPhone/ iTunes	spotify:track:7wgYqjzA3qJLpyz89IlVPA	4/27/07	210	61	40	-7	59	4:14	49	43
That's What I Like	Bruno Mars	iPhone 6/Spotify	spotify:track:0KKkJNfGyhkQ5aFogxQAPU	11/17/16	134	56	85	-5	86	3:27	1	86
The Antidote	Taylor Mcferrin	Laptop/Spotify	spotify:track:4Zd0w81zUn8ZrGthaSc77l	6/9/14	134	75	54	-9	41	3:06	12	32
The Apology Song	Gustavo Santaolalla	Play Music	spotify:track:7nnbb77c4fLnosiTk5QpxQ	9/29/14	94	18	55	-11	34	2:52	91	47
The Bad Touch	Bloodhound Gang	Spotify	spotify:track:5EYdTPdJD74r9EVZBzt	1/1/00	123	74	84	-6	97	4:21	0	68

			qGG									
The Big Three Killed My Baby	The White Stripes	Spotify	spotify:track:3AI7BMmhcoNopWWrmpJPMa	6/15/99	76	97	34	-2	37	2:29	80	36
The Birds	Telefon Tel Aviv	Spotify	spotify:track:4TwMjlyVd6FFqnJyGho73M	1/30/09	120	88	42	-8	18	6:38	6	39
The Brazilian Hipster - Digitally Remastered	Fort Knox Five	Samsung smart phone / Samsung Music App	spotify:track:3sIVBXZR9GSIuFtq5axSmG	4/30/13	105	69	89	-7	67	4:01	23	49
The Chaser	Twin Atlantic	Spotify	spotify:track:6KVLUjhqU7EqDFfD2YqNNO	9/9/16	137	78	46	-6	53	4:06	2	33
The Cross Stands - Live	Worship Central	Spotify	spotify:track:4SVWi4t0ox4kvznj4hnuBz	3/10/13	152	62	24	-6	20	4:49	5	28
The Cup of Life (The Official Song of the World Cup, France '98) - Remix - English Radio Edit	Ricky Martin	iPhone music app	spotify:track:68CzXNGAMmb3k5MhLu0xVb	1/1/98	127	91	71	-7	66	4:40	4	56
The Drums (Din Daa Daa)	Claptone	iPhone 6s	spotify:track:3GxbXFWlq6Qseg4wHJ13qF	3/13/17	122	96	79	-6	72	3:26	4	48
The Eagle and the Hawk	John Denver	ipod	spotify:track:4FU40U6YGW01k1U1lmlnDA	6/17/97	172	45	21	-11	13	3:01	47	34

The Fault Is in Our Stars [Lastufka Remix]	Meghan Tonjes	Spotify	spotify:track:4NHoLJofNYr4Iv2FvLsZpp	8/17/12	95	68	61	-8	28	4:23	3	4
The Feeling	Justin Bieber	Spotify	spotify:track:7vldwy3gdhmPRKWSCi8H61	11/13/15	127	81	53	-6	21	4:05	8	62
The Feeling Begins	Peter Gabriel	iPhone/Spotify	spotify:track:2VrAIjZgqHEX5gP2aW0rpn	5/1/15	114	51	59	-20	16	3:57	10	39
The Fox (What Does The Fox Say?)	Ylvis	Ipod	spotify:track:5HOpkTTVcmZHnthgyxrIL8	9/2/13	128	87	70	-4	55	3:34	11	63
The Greatest	Sia	Spotify	spotify:track:7xHWNBFm6ObGEQPaUxHuKO	10/21/16	192	73	67	-6	73	3:30	1	78
The Hell of It	1927	ITunes	spotify:track:2ma5iFMKDfIn5wy1ldj5q4	8/9/13	100	84	56	-5	62	5:13	1	11
The Imitation Game	Alexandre Desplat	Youtube playlist I made myself	spotify:track:0o9ivTBX7mjTnaUYF4Gk6t	11/7/14	104	31	32	-14	6	2:37	44	63
The Judge	Twenty One Pilots	Google play music	spotify:track:1lguQJjlNrIOoOylYVZN3M	5/15/15	83	80	42	-5	59	4:58	18	73
The Lines In My Hand	Opeth	Spotify	spotify:track:0HkHMZNUw6NS61WWeO6N4X	9/20/11	170	86	42	-10	77	3:49	0	26

The Love I Lost	Harold Melvin & The Blue Notes	iPhone	spotify:track:3oI95Uihdvu7uCiirC1DQA	2/28/95	123	71	68	-11	76	6:24	20	54
The Music Of The Night	Andrew Lloyd Webber	iPod Nano 7	spotify:track:2jF3AQzvTj9L1Ax9Di5BYu	12/10/04	136	8	24	-19	12	5:42	86	54
The Next Step to Regaining Control	empire! empire! (i was a lonely estate)	Ipod shuffle	spotify:track:3Sa8QoQVIo3vQbFGis4yQO	10/23/15	135	68	59	-8	28	6:08	0	22
The Oldest Of Sisters	Balthazar	Spotify	spotify:track:6ggTEdkwzpCISGjsiYODEJ	1/1/12	110	42	65	-8	44	2:44	7	16
The Pretender	Foo Fighters	iPhone	spotify:track:7x8dCjCr0x6x2lXKujYD34	9/25/07	173	96	43	-4	37	4:29	0	77
The Rest Is Noise	Jamie xx	iPhone 7 / Apple Music	spotify:track:7JjzVELpOxqsKP4jNcZXlA	5/29/15	125	66	68	-11	32	4:58	59	49
The Riverboat Song	Ocean Colour Scene	Spotify	spotify:track:1oPg2EYKf2hbS7aDyE8j3S	1/1/01	106	86	48	-6	61	4:59	1	52
The Road	We Are The Ocean	MP3	spotify:track:0oGq3MH2XsD1gDWRBzguRC	1/1/12	153	97	38	-5	35	3:41	0	21
The Spiritual World	Manami Kiyota	Cell Phone										
The Story Of A Starry Night - Remastered	Glenn Miller	Amazon Music on mobile	spotify:track:20fpAGJ7V60nBJ1EyyfH	1/2/96	78	8	29	-14	7	3:29	96	18

			3V									
The Way It Is	Bruce Hornsby	iPhone / Music	spotify:track:6V50MyHPGhEmwYu0Wdyf0t	6/2/86	111	69	58	-12	53	4:58	61	68
The Winner Takes It All	ABBA	iphone	spotify:track:3oEkrIfXfSh9zGnE7eBzSV	1/1/80	126	79	45	-7	52	4:55	57	67
The Wire	HAIM	Iphone	spotify:track:7KdF7Zac5eC9jutk9Qret4	1/1/13	114	63	55	-6	54	4:06	1	61
The Wizard And I - From "Wicked" Original Broadway Cast Recording/2003	Carole Shelley	Spotify	spotify:track:7d9AJSx5MwkUbL48K3ktuA	1/1/04	141	39	37	-8	28	5:10	82	44
The Word - Remastered 2009	The Beatles	Spotify	spotify:track:4GBaPHvAyj4V2jeobD9tsy	12/3/65	121	64	59	-10	79	2:43	1	52
They Live in You	Samuel E. Wright	Spotify on my phone	spotify:track:1SDYD25CrItJZ5nFIa2FxQ	1/1/97	115	26	57	-15	11	3:04	85	45
They Say It's Spring	Blossom Dearie	Ipod	spotify:track:0Gb98vV1mTekDNnUgeeQ6x	11/4/15	111	3	59	-21	40	3:46	93	50
Thieves Like Us	New Order	Spotify	spotify:track:0kQTkXqjtWSPCM2D14cGq3	1/1/94	113	68	66	-6	86	3:58	2	37
Thinking About It - Radio Edit	Just Kiddin	Spotify / iPhone	spotify:track:68kRRV5xUheCkKbk58Ii3j	10/12/14	120	84	69	-6	30	2:53	2	57

This Head I Hold	Electric Guest	Google play music	spotify:track:0LVaveDmmoMEKhBGhVhfoI	4/23/12	162	87	66	-5	96	2:56	3	54
This Is Gospel	Panic! At The Disco	iTunes	spotify:track:3yZQk5PC52CCmT4ZaTIKvv	10/4/13	156	91	57	-5	61	3:07	0	76
This Love	Maroon 5	ipod	spotify:track:6ECp64rv50XVz93WvxXMGF	1/1/02	95	86	71	-5	76	3:26	6	74
This Side Of Paradise	Bryan Adams	Spotify	spotify:track:0V1QAinvloRgY3V6hV3ZO5	1/1/04	106	84	56	-5	51	3:51	9	10
Thomas Tallis 'O Nata Lux de Lumine'	Westminster Cathedral Choir/James O'Donnell	Ipad										
Tighten Up - Pt. 1	Archie Bell & The Drells	Spotify	spotify:track:6IIcvtmuGpWIasqOpyGlyY	1/1/68	125	44	71	-13	72	3:16	24	46
Til the Rivers All Run Dry	The Karaoke Channel	iphone	spotify:track:3UWWRLxRzUb7YDV5F7f05m	6/8/15	117	27	64	-17	48	3:49	53	0
Time Is Running Out	Muse	IPod	spotify:track:2takcwOaAZWiXQijPHIx7B	3/23/04	118	84	59	-6	43	3:57	0	68
Time Table	Kulwinder billa	Phone	spotify:track:2XMHy3TSj4GUP0vZJXo2wB	1/14/15	78	89	70	-2	90	4:44	3	39

Timeless	James Blake	Spotify	spotify:track:49jxn2Mdde8QZUx0AlbD0l	5/5/16	140	32	68	-11	49	4:22	6	45
Times Before	Stockers!	Deezer	spotify:track:2HSVppoug7B9jCZacEN57u	1/1/12	147	62	37	-5	21	4:16	0	9
To Sir With Love	Lulu	iPod nano	spotify:track:1C1dPegkqX6HMoWcAY5LWA	12/29/67	97	34	55	-11	33	2:46	39	46
To The Moon	Lena	iPhone	spotify:track:63bMfwaBfv8vHlU1z3Hx91	1/1/12	102	56	57	-8	49	3:28	50	21
Today and Every Day	Jackie Bond and his Orchestra	iTunes										
Together	CID	Spotify/iPhone	spotify:track:6091uCm2ijbvZRRuyA8P7e	5/6/16	126	98	63	-2	22	3:09	1	44
Tongue Tied	Grouplove	Laptop/Spotify	spotify:track:0GO8y8jQk1PkHzS31d699N	9/2/11	113	94	56	-6	37	3:38	1	77
Too Close	Blue	iPod Touch	spotify:track:5JHbwrC8KTnsxfKkWJx09p	1/1/01	102	76	79	-3	74	3:47	1	41
Torn	Natalie Imbruglia	Iphone	spotify:track:3APayTEWiUl9Ssep4BOXR2	9/5/07	96	93	56	-3	60	4:05	7	71
Touch	Little Mix	Spotify	spotify:track:6oG7bmUn7	11/24/17	102	72	61	-4	55	3:33	3	75

			ws0qYEJ0eEbeG									
Touch	Little Mix	Spotify	spotify:track:6oG7bmUn7ws0qYEJ0eEbeG	11/24/17	102	72	61	-4	55	3:33	3	75
Touch	Little Mix	iTunes	spotify:track:6oG7bmUn7ws0qYEJ0eEbeG	11/24/17	102	72	61	-4	55	3:33	3	75
Touch The Sky	Hillsong United	Spotify	spotify:track:5O2lEvI1K4Fa4KL8vO3e6W	5/26/15	83	43	23	-9	21	4:22	26	61
Trio for Piano, Clarinet and Cello in E-Flat Major, Op. 38 (After Septet Op. 20): V. Scherzo. Allegro molto e vivace – Trio	Ludwig van Beethoven	iTunes	spotify:track:4JbRwIJnQJdZ2dEEUY6G3e	7/20/18	123	20	64	-16	59	3:06	99	26
Tripyra (Bonus Track)	Com Truise	iTunes	spotify:track:7td01NrVI7BzWSIrc8xwAu	1/25/11	164	50	76	-10	48	2:03	12	24
True Colors	Lily Juniper	iPhone Spotify	spotify:track:6cURDHLik2tSwj4oNJdkeZ	3/15/14	171	15	50	-13	34	3:29	92	33
Truly Madly Deeply	Savage Garden	Youtube	spotify:track:69hwHdKl4Y1HusAutt3W6q	3/4/97	168	70	56	-9	82	4:37	40	71
Try A Little Tenderness	The Commitments	Spotify	spotify:track:1gyrCSC99VmnLkkZ08zXMR	1/1/91	111	39	37	-13	10	4:33	1	43

Tush - 2006 Remastered Version	ZZ Top	Ipod classic	spotify:track:6zGDIDjfDkPyNxrEERO3XG	4/18/75	145	89	58	-5	79	2:18	10	64
Twin Size Mattress	The Front Bottoms	Spotify	spotify:track:4AQ4tIKGLXeBQQsybOKTmv	6/2/13	145	87	32	-6	40	4:25	1	48
Two Can Play That Game	Bobby Brown	Spotify on my phone	spotify:track:1gsnWXeNlbfoHpaGRi4oQ0	8/25/92	121	92	61	-7	81	3:34	1	53
Two Little Hitlers	Elvis Costello & The Attractions	iTunes	spotify:track:7MS9MwYlOH3S10Zo4qcF55	1/1/79	114	63	69	-9	77	3:11	2	32
U.N.I.	Ed Sheeran	Spotify	spotify:track:5Ukzlujip1Slqka5OY82YS	9/9/11	74	41	42	-11	58	3:49	32	62
U.N.I.	Ed Sheeran	Spotify	spotify:track:5Ukzlujip1Slqka5OY82YS	9/9/11	74	41	42	-11	58	3:49	32	62
Unconditionally	Katy Perry	Spotify	spotify:track:4fwbGKNExPtPHbor1TBSY4	1/1/13	129	73	40	-5	36	3:49	0	72
Under Cover Names	Peter Hammill	iPod	spotify:track:5i2Hq5E0Kv7xZq094HzDyz	5/17/88	132	73	58	-13	91	4:18	30	1
Under Standing	Eric Lau	Spotify	spotify:track:3zci9PSNoJQXEO7XtIjQR7	3/1/10	96	38	87	-9	81	1:23	0	11

Unfinished Sympathy - 2012 Mix/Master	Massive Attack	Google play music on android phone	spotify:track:0j5FJJOmmnXPd0XajFWkMF	6/10/91	114	84	59	-8	70	5:08	4	60
Unintended	Muse	Spotify	spotify:track:6kyxQuFD38mo4S3urD2Wkw	1/1/99	139	28	49	-12	16	3:57	65	58
Up (feat. Demi Lovato)	Olly Murs	Iphone Music	spotify:track:3Sj8cecsigmHukuSILzDkg	7/1/16	115	81	69	-6	65	3:43	2	26
Up All Night	ARTY	Samsung S7	spotify:track:1tUiPKYOob0YbMdVRbr79w	10/9/15	130	84	52	-5	52	4:07	1	48
Uprising	Muse	Mp3	spotify:track:4VqPOruhp5EdPBeR92t6lQ	9/10/09	128	91	60	-4	41	5:05	0	76
Valierie ('58 Version)	Amy Winehouse	iTunes										
Valley	Perfume Genius	Spotify	spotify:track:0EScPNUSasv4VbXyLZKgSG	5/5/17	100	25	75	-9	44	3:10	81	53
Vamp	Graham Fitkin Band	iPod										
Vicar In A Tutu - 2011 Remastered Version	The Smiths	iPod	spotify:track:59cZGLKHgcYWd0z4fvtikE	1/1/86	125	88	50	-7	81	2:24	0	45
Video Games - Remastered	Lana Del Rey	Music (IPhone)	spotify:track:0fBSs3fRoh1yJcne77fdu9	1/1/12	73	25	24	-10	18	4:42	81	70

Vincent	Don McLean	Spotify	spotify:track:0VNzEY1G4GLqcNx5qaaTl6	1/1/71	92	9	42	-19	46	4:03	91	64
VinterNoll2	kent	Spotify	spotify:track:7sgGULtAhIts3SeZdou7py	11/4/02	138	95	50	-4	43	4:25	0	45
Violin Concerto No.5 In A, K.219: 3. Rondeau (Tempo di minuetto)	Wolfgang Amadeus Mozart	Spotify	spotify:track:5UA4wgE9BzWYw3pllKCsO2	2/9/14	133	8	37	-22	29	8:55	96	18
Virtual Paradise	AK	Mobile phone / Google play music	spotify:track:1qukC9Q7L3kBUBNrG3pYii	11/17/15	174	83	59	-5	4	3:30	65	42
Voaría A Compostela	Milladoiro	Spotify	spotify:track:0TaiqzrFKJf75f41rO3ZqL	1/1/05	90	14	47	-18	30	3:59	81	5
Wait For It	Leslie Odom Jr.	Spotify	spotify:track:7EqpEBPOohgk7NnKvBGFWo	9/25/15	87	47	56	-10	51	3:14	13	69
Waiting for a Star to Fall	Boy Meets Girl	Spotify	spotify:track:5suJZTE8SpSmR4zMqxLg3V	2/26/10	116	73	66	-10	59	4:26	9	58
Wake Me Up	Boyce Avenue	Spotify	spotify:track:7Dij8mBegb8uhckk1TRJUz	4/28/15	100	21	45	-10	37	3:24	84	53
Wake Me up Before You Go-Go	Wham!	Sonos	spotify:track:0ikz6tENMONtK6qGkOrU3c	10/23/84	82	57	62	-12	90	3:51	27	79

Wake the Dead	Comeback Kid	iPod	spotify:track:1i8IMyKJZx7QypOnfyrytu	1/1/05	108	96	45	-4	35	3:17	0	48
Walking In Your Footsteps - Remastered 2003	The Police	Itunes	spotify:track:0UD8XYV7rmmrOHGhhFQwkE	6/1/83	197	53	61	-15	95	3:36	92	36
Wannabe - Radio Edit	Spice Girls	Phone/Amazon Prime Music	spotify:track:1Je1IMUlBXcx1Fz0WE7oPT	11/4/96	110	86	77	-6	89	2:53	10	81
Watch Me	Tempest	Sportify	spotify:track:61Es9dnL8KNMOFtxc6RMWB	6/24/16	75	98	69	0	32	3:12	1	6
Waterbed	The Chainsmokers	iPhone/Music App	spotify:track:5glABIjb1lnJ2zLl3RxQEI	10/23/15	105	87	61	-7	17	3:30	0	55
Waverley Steps	Roddy Woomble	iPhone	spotify:track:3fBnLBjhE0elUfCI6GKOou	1/1/06	157	54	29	-5	15	4:08	9	17
We Are Bulletproof Pt.2	BTS	Spotify	spotify:track:2Kspa0jD2MSkaEdonf4Cnb	6/12/13	144	95	75	-5	87	3:45	1	68
We Are Young (feat. Janelle Monáe)	fun.	Spotify	spotify:track:5rgy6ghBq1eRApCkeUdJXf	2/14/12	184	64	38	-6	74	4:11	2	72
We Care A Lot	Faith No More	Desktop	spotify:track:2z1uVu3OwTmPlfqt5TWk5k	4/23/87	109	89	80	-6	90	4:04	8	47
We Dance (Live)	Steffany Gretzinger	Laptop	spotify:track:2oq908qDm	4/21/14	72	28	29	-10	38	4:42	95	56

			ARqhOlqVvEUbJ									
We Didn't Start the Fire	Billy Joel	iPhone/Spotify	spotify:track:38bDGWuyYdSdNfrFfbCiVS	10/2/01	145	97	71	-5	89	4:48	8	60
We Know The Way - From "Moana"	Opetaia Foa'i	ipod / iTunes	spotify:track:3JlqQyrjz3MtZk2AK5V57u	11/11/16	98	59	69	-9	38	2:21	35	54
What Do You Mean?	Justin Bieber	Spotify	spotify:track:3pzjHKrQSvXGHQ98dx18HI	11/13/15	125	57	85	-8	79	3:26	59	76
What Is Love	Haddaway	ipod/ iTunes	spotify:track:2ahnofp2LbBWDXcJbMaSTu	1/1/93	124	77	68	-8	74	4:30	2	72
What's The Frequency, Kenneth?	R.E.M.	Spotify	spotify:track:1yStBw3HI2pnjbO3ihq33N	1/1/94	95	91	40	-5	69	4:00	18	53
When Adam Fell, Op. 89	Alexander Goehr	iPod	spotify:track:3PoCrk4KhQhZVqm12a19hU	2/5/13	116	3	29	-25	4	15:27	98	9
When I	DJ Zinc	Spotify	spotify:track:4aDcXmuGJWeRdOpFf6Rc0I	12/21/16	127	93	76	-4	66	4:04	1	38
When The Sun Goes Down	Arctic Monkeys	iTunes	spotify:track:2zzLRQ78kKfPTx8FJQCdC2	1/29/06	169	88	35	-5	41	3:22	3	71
When We Were On Fire	James Bay	Spotify	spotify:track:5mY6VWwXebTz2ogby	3/23/15	114	81	69	-5	75	3:58	0	47

			LOlhF									
When We Were Young	Adele	SPOTIFY	spotify:track:0cj2joJcY6b4XSRfj2eZOl	11/20/15	144	60	38	-6	26	4:51	30	75
When Will My Life Begin? - From "Tangled"	Mandy Moore	iTunes (on laptop)	spotify:track:4WOTwgkLN0puzCVg2WnV2N	6/22/18	108	42	68	-8	43	2:32	12	53
Where Is Your Rider	The Oh Hellos	Apple Music	spotify:track:3kwDAannUD2Q7Z0bnPdXgO	10/16/15	100	46	65	-11	44	3:19	34	38
Wherever You Will Go	The Calling	Play Music	spotify:track:2n6FX3Jcg4b4Leoz0GOqBF	1/1/01	112	72	56	-5	37	3:29	4	68
Whipsnade	Suede	iTunes	spotify:track:0os9ywgdJQ3Q10u46WwJeB	10/6/97	113	72	55	-9	48	4:22	64	15
Whiskey, Whiskey, Whiskey	John Mayer	iphone	spotify:track:50YQFbfIVk2XMnVFKAuuib	5/18/12	113	42	41	-9	19	4:39	29	55
Whiskey, Whiskey, Whiskey	John Mayer	Itunes	spotify:track:50YQFbfIVk2XMnVFKAuuib	5/18/12	113	42	41	-9	19	4:39	29	55
White Lines	Danny Brown	Iphone	spotify:track:3fqfuK6PQITWU0fCKfo31d	9/27/16	154	86	41	-5	47	2:24	21	42
White Waking	Les Rallizes Dénudés	Ipod Classic	spotify:track:2d1lDxWzz4L1D3Rk1Le	8/14/17	142	27	37	-13	19	4:53	97	20

			DR0									
Whose Bed Have Your Boots Been Under? - Live	Shania Twain	IPOD	spotify:track:3CAjCIp6vqAYutYtyLtaSe	3/3/15	132	91	59	-5	74	4:46	0	36
Why'd You Only Call Me When You're High?	Arctic Monkeys	Apple Music	spotify:track:086myS9r57YsLbJpU0TgK9	9/9/13	92	63	69	-6	80	2:41	5	75
Winning	The Sound	Spotify on my PC	spotify:track:7EOCSmPvQ8psymuLTsKfN2	1/1/81	151	72	43	-9	25	4:18	0	40
Won't Be Afraid	The Cat Empire	Spotify	spotify:track:1JREvL53b64WwKUBzZTgyq	1/1/07	88	79	63	-5	97	4:11	5	27
Wonder (Live) [Spontaneous]	Amanda Cook	Spotify	spotify:track:390Wrd097A2Fa15NzxukHa	4/21/14	148	27	21	-10	8	6:32	38	52
Wonderful World	James Morrison	Spotify	spotify:track:2W2mN3qRAvF172TlNdr71e	1/1/06	79	71	48	-5	59	3:30	6	52
Wonderland	Taylor Swift	Apple Music	spotify:track:6RvRzl1YJTDnUvdOtV21IK	1/1/14	184	69	42	-5	20	4:06	4	53
Wonderwall	Oasis	Spotify	spotify:track:79RUMZfMNMpqZnswovvTqv	10/2/95	174	84	41	-6	46	4:19	0	74
World News	Local Natives	Spotify	spotify:track:0yeCTb6RJGwWReSjq04	11/2/09	122	81	54	-9	56	4:32	1	32

			0o1									
Worried About Ray	The Hoosiers	Ipad	spotify:track:5WxDUKPTu3SDBma67yf3H5	10/22/07	174	81	49	-4	60	2:47	0	49
Wrapped Up	Olly Murs	spotify	spotify:track:6nM9Sr6MPcfpqwQTD7PZGi	11/21/14	122	85	79	-5	95	3:06	9	64
Wrecking Crew	To Kill A King	Spotify	spotify:track:7JWDwgUIlZiVLeOdspKF7c	10/16/11	54	23	31	-15	32	3:55	74	13
Ya Veran	QUITAPENAS	iPhone 6	spotify:track:5ayp2hoKsB2nQiDb3hvS0J	3/31/17	133	84	65	-8	72	3:36	12	21
You & Me	Marc E. Bassy	Spotify	spotify:track:7yCqehT9dKACTFy7YugP0J	10/13/17	85	63	70	-8	63	3:38	5	66
You Are My Sunshine	Max Abrams	Spotify	spotify:track:6qYS1thYj5jvX0PeViia6e	2/26/14	116	27	66	-11	56	2:58	35	1
You Are So Beautiful	Jools Holland	ipod	spotify:track:3oZXYW7e7s0VzIAVmtyVyN	12/4/15	135	46	45	-10	35	3:26	64	15
You Can Call Me Al	Paul Simon	Spotify	spotify:track:0qxYx4F3vm1AOnfux6dDxP	8/12/86	128	76	78	-8	82	4:40	18	73
You Can't Always Get What You Want	Band From TV	Spotify	spotify:track:0OTi6ShQu71aE4Kh2uywwS	1/1/07	176	62	55	-7	67	4:24	15	28

You Don't Know Love	Olly Murs	iPhone	spotify:track:3Iyzdie1LZW22Z9S2hIvHl	7/8/16	119	66	68	-6	37	3:18	0	58
You Don't Know Me - Radio Edit	Jax Jones	Apple music	spotify:track:1rFMYAZxBoAKSzXI54brMu	12/9/16	124	66	88	-6	69	3:34	16	75
You Give Love A Bad Name	Bon Jovi	Spotify	spotify:track:3i17RhgJVzIHoXVoqybyYs	1/1/94	123	95	56	-4	81	3:44	7	67
You Wanna Freak Out	My Morning Jacket	Ipod	spotify:track:6QgC9zegBJQc2dvZxLr3eS	6/6/11	153	87	43	-7	79	3:20	1	21
You, Visionen im Speigel	Yuzuki	iTunes										
You'll Lose A Good Thing	Barbara Lynn	iTunes	spotify:track:5CEREcGR5WaLt40YzTQ62e	1/1/63	104	32	59	-13	62	2:40	61	48
You're Gorgeous	Babybird	iPhone	spotify:track:0yJiIt4icur8txxfLRDJZK	7/16/04	124	69	57	-7	26	3:42	24	52
You're My Excuse to Travel	Baths	iPod	spotify:track:1qhcbl8f8GMR4om9UlPzlC	7/6/10	95	82	50	-6	38	3:35	21	30
You're Welcome	Dwayne Johnson	Phone	spotify:track:6U4VqEHy4n5VeiH4pQPL24	11/18/16	135	75	78	-7	69	2:44	28	76
You’re Dead To Me	Blitz Kids	Spotify	spotify:track:0G9rkqGhyi8BDGDYBx3JvC	1/1/12	170	96	34	-6	38	3:50	0	5

Young Americans - 2016 Remastered Version	David Bowie	I-phone	spotify:track:7uPmQttafLiJyju14JREY4	3/7/75	84	76	61	-9	89	5:14	17	61
Young Girl	Gary Puckett & The Union Gap	Ipod Nano	spotify:track:3F64pIPqvcujnwvcVLVZ64	1/1/68	119	54	41	-8	45	3:14	65	55
Zadok The Priest (Coronation Anthem No.1, HWV 258)	George Frideric Handel	iPod Nano 7	spotify:track:4tDs2Df4vjFqN0twseXB9t	1/1/13	96	17	39	-15	14	5:16	90	38
Zero	Melt-Banana	iTunes	spotify:track:7ddCej9llJ7YRo1uGvM0uk	10/1/13	131	91	41	-4	18	3:45	11	31
リサフランク 420 / 現代のコンピュー	MACINTOSH PLUS	soundcloud										
